# Phase segregation and nanoconfined fluid O_2_ in a lithium-rich oxide cathode

**DOI:** 10.1038/s41563-024-01873-5

**Published:** 2024-05-13

**Authors:** Kit McColl, Samuel W. Coles, Pezhman Zarabadi-Poor, Benjamin J. Morgan, M. Saiful Islam

**Affiliations:** 1 Department of Chemistry, University of Bath, Bath, UK; 2 The Faraday Institution, Harwell Science and Innovation Campus, Didcot, UK; 3 Department of Materials, University of Oxford, Oxford, UK

**Keywords:** Batteries, Atomistic models, Computational chemistry

## Abstract

Lithium-rich oxide cathodes lose energy density during cycling due to atomic disordering and nanoscale structural rearrangements, which are both challenging to characterize. Here we resolve the kinetics and thermodynamics of these processes in an exemplar layered Li-rich (Li_1.2–*x*_Mn_0.8_O_2_) cathode using a combined approach of ab initio molecular dynamics and cluster expansion-based Monte Carlo simulations. We identify a kinetically accessible and thermodynamically favourable mechanism to form O_2_ molecules in the bulk, involving Mn migration and driven by interlayer oxygen dimerization. At the top of charge, the bulk structure locally phase segregates into MnO_2_-rich regions and Mn-deficient nanovoids, which contain O_2_ molecules as a nanoconfined fluid. These nanovoids are connected in a percolating network, potentially allowing long-range oxygen transport and linking bulk O_2_ formation to surface O_2_ loss. These insights highlight the importance of developing strategies to kinetically stabilize the bulk structure of Li-rich O-redox cathodes to maintain their high energy densities.

## Abstract (web summary)

Lithium-rich oxygen-redox cathodes demonstrate high capacities, but lose energy density when cycled, showing cation disordering and formation of nanovoids and bulk molecular O_2_. These structural changes are shown to be a consequence of a kinetically viable and thermodynamically favoured local phase segregation mechanism.

## Main

Lithium-ion batteries using conventional cathodes based upon layered LiCoO_2_ have revolutionized portable electronics and electric vehicles. Yet the continuing demand for improved battery performance means further increases in energy density are needed. Lithium-rich oxide cathodes offer higher energy densities than conventional cathodes because they utilize capacity from both transition metal ion and oxygen redox when cycled^1,2^. Oxygen redox is typically accompanied by bulk structural changes associated with a large loss of energy density^3,4^. Understanding these structural changes and their relationship to O-redox behaviour is one of the major challenges for improving Li-rich cathodes. While some aspects of these O-redox-driven structural changes are already understood, such as the involvement of transition metal migration^5,6^ and oxygen dimerization^7,8^, the atomic- to nanoscale picture remains incomplete, in part due to challenges in experiments and modelling to characterize the structure and O-redox behaviour of Li-rich cathodes during cycling^9^.

Electronic structure modelling is a powerful tool for understanding atomic-level structures and predicting redox behaviour^10^. Modelling O redox, however, is non-trivial^9,11^. Computational predictions of O-redox behaviour depend on the choice of structures used in these models. Because O-redox cathodes undergo atomic rearrangements during cycling, after the early stages of the first charge, the structures of these cathodes are not known a priori and must be solved in silico. For computational modelling studies to make useful predictions of O-redox behaviour, both the kinetics and thermodynamics of structural rearrangements must be considered. Firstly, structural rearrangements must proceed via kinetically accessible paths. Secondly, any kinetically accessible structures should be stable with respect to further rearrangement on relevant experimental timescales. Computational approaches that predict behaviour using structures that form only via kinetically inaccessible pathways or that are kinetically unstable to further rearrangement can yield unrealistic descriptions of O redox. Finally, to understand cathode behaviour over multiple cycles, it is necessary to consider thermodynamic factors. O-redox cathodes cycled towards a thermodynamic ground state increasingly exhibit both crystallographic site disorder^12,13^ and nanoscale structural changes, such as the formation of nanovoids^14–17^. To model crystallographic disorder and nanoscale structural features, computational studies must search a vast configurational space to identify stable low-energy configurations while also using cell sizes large enough to capture relevant structural features. Many previously proposed O-redox mechanisms are based on computational studies that assessed structures using density functional theory (DFT)^18–27^. However, DFT is too computationally expensive to directly investigate disorder and nanoscale structures, meaning that additional modelling methods are required to provide a complete picture of O-redox behaviour.

Here, we use a computational strategy that directly addresses these kinetic and thermodynamic factors in O-redox modelling. We have used long-timescale ab initio molecular dynamics (AIMD) to identify kinetically viable atomic-scale rearrangements during the first charge and, in parallel, have developed a DFT-derived cluster expansion model of oxygen redox that accounts for disorder and nanoscale structural changes produced after many cycles, which we have used to perform large-scale Monte Carlo simulations. This approach allows us to efficiently search the vast configurational space for low-energy structures at the top of charge and to conduct this search in structures containing ~50,000 atoms to resolve thermodynamically driven nanoscale structural rearrangements.

We have applied this combined strategy to high-capacity O2-layered Li_1.2–*x*_Mn_0.8_O_2_, which is an exemplary system for understanding Li-rich oxide cathodes. We identify a kinetically viable O-redox mechanism in which the formation of transient interlayer superoxide and peroxide intermediates drives out-of-plane Mn migration, resulting in O_2_ molecules forming within the bulk structure. The thermodynamic ground state structure at the top of charge exhibits phase segregation into a two-phase mixture of MnO_2_ and O_2_. Bulk O_2_ molecules are confined within nanometre-sized Mn-deficient voids that form a connected percolating network. These O_2_ molecules have a nanoconfined supercritical fluid character and can potentially diffuse through the network of voids, providing a mechanistic link between bulk O_2_ formation and surface O_2_ loss.

## Kinetics of structural change: charged O2-Li_*x*_[Li_0.2_Mn_0.8_]O_2_

**Fig. 1:**
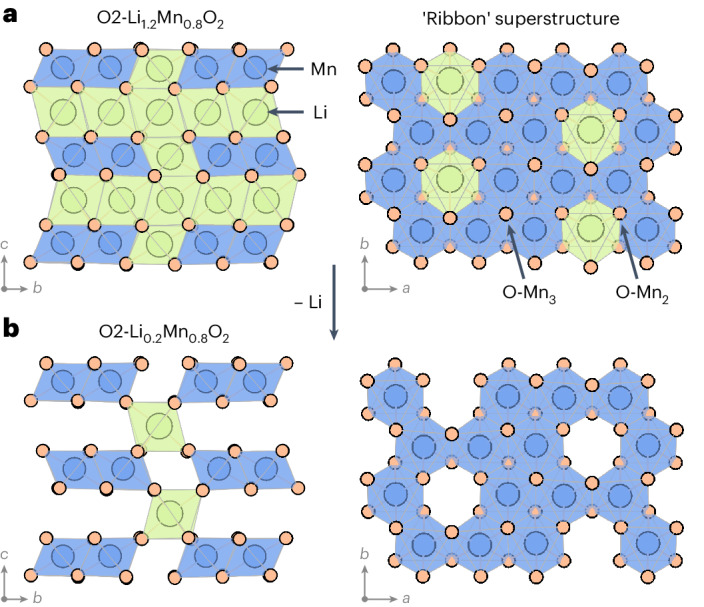
Structures of pristine O2-Li_1.2_Mn_0.8_O_2_ and metastable O2-Li_0.2_Mn_0.8_O_2_. **a**, Pristine O2-Li_1.2_Mn_0.8_O_2_ showing the octahedral interlayer Li sites, the ‘ribbon’ superstructure arrangement of lithium ions in the Mn layer and the two types of oxygen coordination: O–Mn_2_ and O–Mn_3_. **b**, Metastable O2-Li_0.2_Mn_0.8_O_2_ with the ribbon structure preserved, obtained in the absence of host framework rearrangements, showing octahedral lithium ions remaining in the Li layer only in sites directly above and below the vacancies in the Mn layers. Green and blue shading shows the connectivity around LiO_6_ and MnO_6_ octahedra, respectively.

The crystal structure of lithium-rich O2-Li_1.2_Mn_0.8_O_2_ (Fig. 1a) features O2-stacked^28^ layers of octahedrally coordinated Li and Mn^29,30^. A regular pattern of Li sites in the Mn layers gives a characteristic ‘ribbon’ superstructure^31^. Oxygen ions are coordinated to three (O–Mn_3_) or two (O–Mn_2_) Mn atoms, with the O–Mn_2_ atoms coordinated to the Li ions in the Mn-rich layers. These O–Mn_2_ atoms have a single O 2*p* orbital at the top of the valence band that is unhybridized with any Mn 3*d* orbitals and is susceptible to oxidation upon charge^19,32^.

To understand the first-cycle behaviour of O2-Li_1.2_Mn_0.8_O_2_ in the oxygen-redox regime, we modelled charging the pristine cathode structure past the conventional transition metal redox limit (that is, Mn^4+^) by removing one lithium ion per formula unit, giving a composition of Li_0.2_Mn_0.8_O_2_. When relaxed using DFT, this delithiated structure appears stable with respect to structural rearrangement, with no observed Mn rearrangement or oxygen dimerization (Fig. 1b). This absence of structural rearrangements is an artefact of this DFT relaxation approach, which yields a structure trapped in a local minimum. This DFT-relaxed delithiated structure, however, is unstable with respect to structural rearrangement over experimental timescales^33,34^.

To identify kinetically accessible structural rearrangements, we performed high-temperature AIMD, analogous to a thermal decomposition experiment, by heating metastable Li_0.2_Mn_0.8_O_2_ to 900 K and holding it at that temperature for 400 ps (Methods)^35,36^. Using AIMD gives an unbiased search for structural rearrangements that selects for kinetically viable rearrangement pathways. For structures obtained from this AIMD trajectory that displayed oxygen dimerization or transition metal ion migration, we performed full relaxations (quenching) using higher-accuracy hybrid DFT calculations to evaluate whether these rearrangements are thermodynamically favoured.

**Fig. 2:**
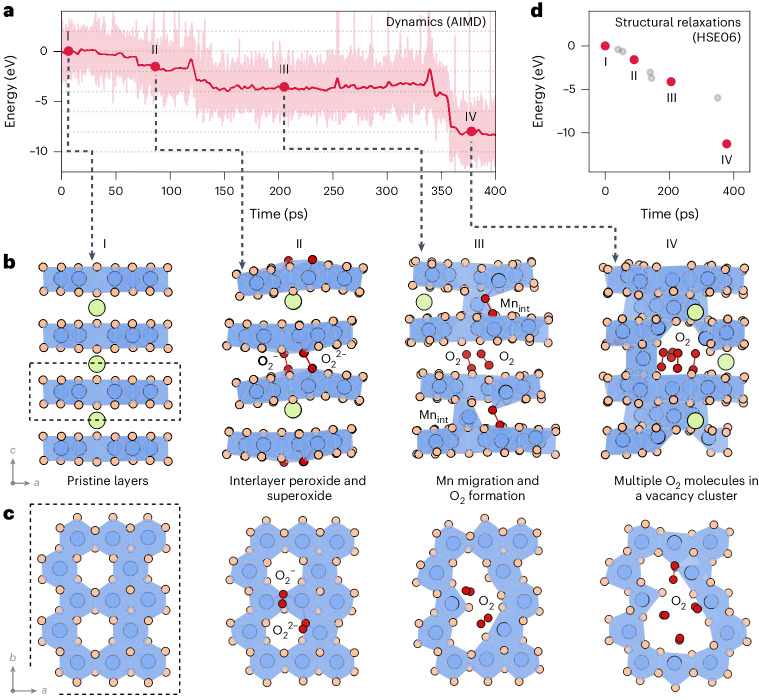
Mechanism of O–O dimerization and Mn migration in Li_0.2_Mn_0.8_O_2_. **a**, The change in total energy from 400 ps DFT + *U* AIMD simulations at 900 K, with indicated positions showing structures that were extracted for further analysis. The shaded region shows the range of the fluctuations of total energy, and the red line indicates the energy averaged with a 1 ps time window. **b**,**c**, Optimized HSE06 equilibrium geometry of the extracted structures from **a** in both the *a*–*c* (**b**) and *a*–*b* planes (**c**). Some lithium ions have been removed for clarity. **d**, The change in the total energy for HSE06 geometry relaxations of structures I–IV plotted as a function of the AIMD simulation time. Red dots correspond to the structures in **b** and **c**, with grey dots indicating additional relaxed structures (Methods). Green, blue and orange circles show Li^+^, Mn and lattice O ions, respectively. Red circles show O atoms in O–O dimers. Blue shading shows the MnO_6_ octahedra.

Applying this procedure to delithiated O2-Li_0.2_Mn_0.8_O_2_ (Fig. 2a) reveals a previously unreported O-redox mechanism. O ions in O–Mn_2_ sites are preferentially oxidized, and interlayer peroxide (O_2_^2–^) and superoxide (O_2_^–^) species then form from these O–Mn_2_ sites in adjacent transition metal layers (Fig. 2b,c, structure II). Once these O atoms have dimerized, their bonding interaction with their neighbouring Mn ions is reduced (Supplementary Fig. 4). This drives Mn migration into the interlayer space, proceeding through hops to first or second neighbour sites. This Mn migration results in de-coordination of the O–O dimers from Mn to form a pair of O_2_ molecules (structure III). Over longer timescales (~400 ps), six Mn ions migrate to the interlayer space, and more O_2_ molecules form, which group together in the Mn vacancy cluster (structure IV). This process is kinetically viable and causes an overall thermodynamic stabilization of the system, with structure IV approximately 11 eV per cell more stable than the starting structure I (Fig. 2d). O–O dimerization requires an interlayer distance between O atoms of <1.5 Å. This is achieved by a rippling of the transition metal layers that gives a local contraction of the space between them, even while the average interlayer spacing and *c* lattice parameter are not substantially changed (Supplementary Table 1 and Supplementary Fig. 3b).

Previous studies have proposed an alternative pathway to forming O_2_ in Li-rich layered cathodes, in which Mn migration occurs before oxygen dimerization^21,25^. Our results show no evidence of this mechanism in Li_0.2_Mn_0.8_O_2_. Indeed, our AIMD simulations consistently demonstrate interlayer O–O dimerization before Mn migration (Supplementary Fig. 2). Mn migration to interlayer sites without accompanying oxygen dimerization gives structures that are higher in energy than structures that contain interlayer superoxide and peroxide species (Supplementary Fig. 3). These results, therefore, indicate that the kinetically favoured first step in the O-redox mechanism is interlayer O–O dimerization rather than Mn migration.

Transition metal migration and structural degradation in Li- and Mn-rich cathodes have previously been attributed to lattice strain between Li_2_MnO_3_ and LiTMO_2_ domains^37^ (where TM is Ni or Co). Our results, described here for the single-phase ribbon-structured Li_1.2–x_Mn_0.8_O_2_, suggest that there is a driving force for both transition metal migration into the Li layer and structural degradation, irrespective of whether nanodomains are present. Structural degradation is initiated by oxidized framework O atoms and proceeds if the transition metal interlayer separation can contract to permit interlayer O–O dimerization. Consequently, preventing interlayer O–O dimerization is probably an effective strategy for improving the kinetic stability of the transition metal layers against rearrangement. This might be achieved by increasing the spacing between layers with ‘pillaring’ cations or by including large interlayer ions, such as for Na-ion cathodes^38^.

## Mn-deficient nanovoids and O_2_ confinement

**Fig. 3:**
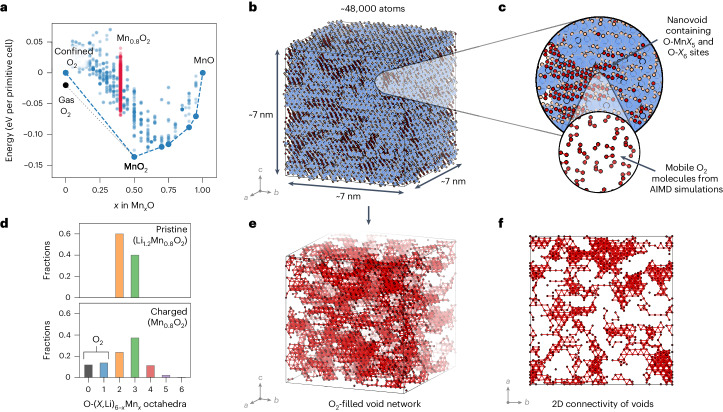
Local phase segregation and formation of Mn-deficient O_2_-filled nanovoids at the top of charge. **a**, The DFT-calculated convex hull of formation energies used to fit the cluster expansion along the O_2_–MnO_2_–MnO tie line, showing the position of structures with the delithiated cathode composition (Mn_0.8_O_2_) above the ground state hull. **b**, A supercell of 48,000 atoms obtained from canonical lattice Monte Carlo simulated annealing of Mn_0.8_O_2_. Lattice O–Mn*X*_5_ or O–*X*_6_ sites, where *X* is a cation vacancy, represent O_2_ molecules (Supplementary Note 1). **c**, The detailed lattice structure of a section from **b**, showing O_2_-filled void regions arising from clustered O–Mn*X*_5_ and O–*X*_6_ sites. The inset shows the relaxed structure of a box of confined O_2_ molecules. **d**, The analysis of the O coordination environments in pristine Li_1.2_Mn_0.8_O_2_ and delithiated Mn_0.8_O_2_ after charge. Approximately 20% of the O atoms after cycling are O_2_ molecules (that is, O–Mn*X*_5_ or O–*X*_6_) (Supplementary Note 1). **e**, A 3D representation of the void network, showing the O atoms in O_2_ molecules only. **f**, A 2D slide of the percolating void network, highlighting the bottlenecks between voids.

The AIMD simulations above investigate kinetically viable structural rearrangements during the first charge but do not permit enough Mn migration to obtain a thermodynamic ground state structure. To identify thermodynamically stable structures at the top of charge (Mn_0.8_O_2_) that are representative of the cathode after many cycles, while accounting for disorder and nanoscale structural changes, we developed a cluster expansion model of oxygen redox. For this cluster expansion model, we computed the energies of structures along the O_2_–MnO_2_–MnO tie line using hybrid DFT. We consider two situations for the O_2_ end-member: free gaseous O_2_, which corresponds to oxygen lost from the cathode, and O_2_ molecules confined in the bulk (Supplementary Notes1 and 2), and construct a convex hull of formation energies (Fig. 3a), which defines the ground state structures at a given composition. Structures at the top of charge (Mn_0.8_O_2_) are all above the ground state hull connecting the O_2_ states and MnO_2_, indicating that Mn_0.8_O_2_ is metastable with respect to decomposition into a two-phase mixture of MnO_2_ and O_2_. The gaseous O_2_ state is below the confined O_2_ state; that is, the lowest energy product is for O_2_ to be lost from the system. Importantly, Mn_0.8_O_2_ is above the ground state hull even for O_2_ molecules confined in the bulk. This indicates that Mn_0.8_O_2_ is thermodynamically susceptible to decomposition into MnO_2_ and O_2_, even if these O_2_ molecules are trapped in the bulk and cannot be lost from the system.

To represent the bulk structure accurately, we fitted our cluster expansion using the energy of confined O_2_. We developed a sampling strategy that established that off-lattice O_2_ molecules can be represented by O–Mn*X*_5_ and O–*X*_6_ sites in a lattice model, where *X* is a cation vacancy (Supplementary Note 1). To systematically search for low-energy structures and examine nanoscale features, we used this cluster expansion model to run lattice Monte Carlo annealing simulations of structures containing 48,000 atoms, which is several orders of magnitude larger than is possible using pure DFT (Fig. 3b). Our simulations predict local phase segregation-type behaviour with the formation of MnO_2_-rich regions and Mn-deficient nanovoids that contain confined O_2_ molecules (Fig. 3c). Approximately 20% of the O atoms in the system form O_2_ molecules. O_2_ molecules formed at the surface will be lost immediately, as this is the thermodynamically lowest energy pathway. However, O_2_ molecules formed in the bulk will be trapped if they cannot escape the system. The phase segregation also results in disorder (Fig. 3d) and a complete loss of the layered structure.

The O_2_-filled nanovoids vary in size from ~0.5 nm to >1.5 nm in length and form a three-dimensional (3D) percolating network connecting 90% to 95% of the O_2_ molecules. This percolating network may permit O_2_ transport through the cathode but is highly tortuous, with a mean microscopic tortuosity factor *τ* of *~*24, compared with *τ* = 1 for the pristine structure. The structure can be characterized as multiple large voids filled with many O_2_ molecules, often connected by narrow passages, with these likely to act as bottlenecks for any potential oxygen transport (Fig. 3e,f).

## Dynamics of nanoconfined O_2_ molecules

**Fig. 4:**
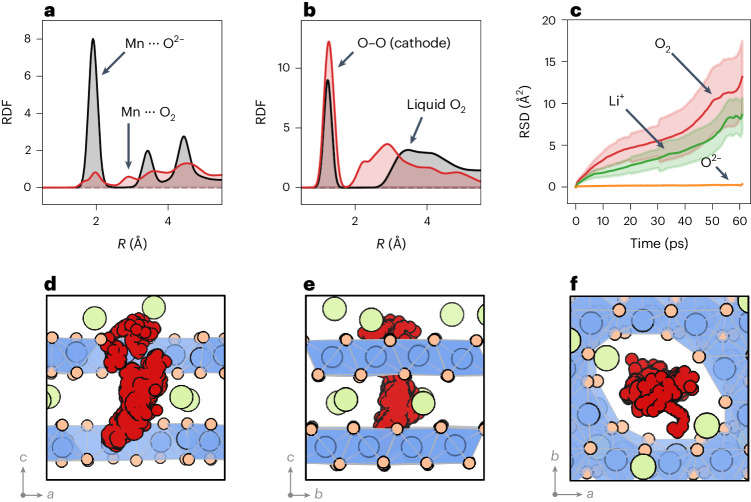
Room temperature transport and structural properties of O_2_ molecules in nanovoids. **a**, The RDF of Mn⋯O^2–^ and Mn⋯O_2_ species in Li_0.2_Mn_0.8_O_2_ from AIMD simulations at 300 K, showing a solid-like character for the O^2–^ lattice and a fluid-like character for the O_2_. **b**, The RDF of O⋯O species, showing the compressed character of the O_2_ in the cathode relative to liquid O_2_ at ambient pressure. **c**, The MSD of O_2_ molecules, Li^+^ ions, and O^2–^ ions showing O_2_ and Li^+^ diffusion. The shaded region shows the estimated standard deviation in the MSD. **d**–**f**, The trajectory of a single O_2_ molecule over 60 ps shown along the three different crystallographic axes; red circles show the position of the O atoms in the O_2_ molecule every 200 fs. The O_2_ molecule moves between layers, indicating that oxygen diffusion is possible through stacked voids. Green, blue and orange circles show Li^+^, Mn and lattice O ions, respectively. Blue shading shows MnO_6_ octahedra.

Having established that the cathode bulk structures at the top of charge feature nanovoids that contain O_2_ molecules, we performed additional molecular dynamics simulations at 300 K to characterize the room temperature dynamics of these O_2_ molecules. The resulting radial distribution functions (RDFs) for Mn and lattice O^2–^ pairs show sharp peaks, indicative of an ordered crystalline solid (Fig. 4a). By contrast, the Mn–O_2_ RDF shows broader peaks, with non-zero values between the peaks, indicating that the molecular O_2_ is mobile. A similar effect is seen in the RDF for the O atoms of only the O_2_ molecules (Fig. 4b). The second neighbour peak describes the O_2_ inter-molecular distance. For solid crystalline O_2_, this second peak in the RDF would be sharp; instead, we observe a broad second peak consistent with these O_2_ molecules exhibiting rotational and translational degrees of freedom.

Figure 4b compares the RDF for O_2_ in the cathode bulk with a simulated RDF of liquid O_2_ under ambient conditions (Fig. 4b). The cathode bulk O_2_ has a second-neighbour peak maximum at a much smaller distance than for liquid O_2_ and has a density of approximately 1.45 g cm^–3^. This density is higher than that of liquid O_2_ (1.141 g cm^–3^ at *T* = 88 K and ambient pressure) but lower than the density of solid β-O_2_ (2.21 g cm^–3^ at *T* = 299 K and 5.5 GPa)^39^. The critical point for O_2_ is at 154.6 K and 5.05 MPa, so O_2_ in the cathode bulk is predicted to be in the supercritical regime.

The mobile character of O_2_ and the presence of a percolating void network mean that the O_2_ might diffuse through the structure. To probe possible room temperature O_2_ transport, we ran AIMD at 300 K on a Li_0.2_Mn_0.8_O_2_ system with percolating Mn void pathways containing six O_2_ molecules (Fig. 4d–f). Figure 4c shows the mean squared displacements (MSDs) calculated from this simulation for the O_2_ molecules, lattice O^2–^ ions and Li^+^ ions. The MSD for the lattice O^2–^ ions shows little change from the initial value of zero (Fig. 4c), indicating that O^2–^ ions are not diffusing, as expected. In contrast, the MSDs for the O_2_ molecules and Li^+^ ions increase with time, indicating substantial diffusion of both species. The calculated diffusion coefficients for O_2_ molecules and Li^+^ ions are 1 × 10^–7^ cm^2^ s^–1^ and ~7 × 10^–8^ cm^2^ s^–1^, respectively, indicating that the oxygen molecules are highly mobile over this short length scale. The non-crystalline diffusive character of this bulk O_2_ means that these O_2_ molecules can be considered a high-density nanoconfined fluid. Figure 4d–f shows the positions of the O atoms from a single O_2_ molecule throughout the AIMD simulation. The molecule crosses the interlayer space and moves from one layer to beyond the layer above, demonstrating the potential for long-range O_2_ diffusion given sufficient connectivity between voids.

## Atomic to nanoscale mechanisms of oxygen redox

**Fig. 5:**
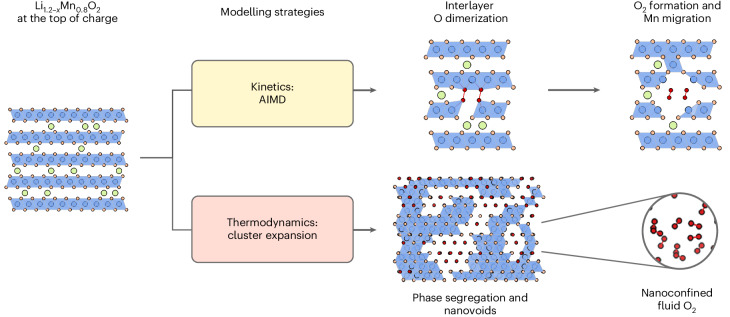
Schematic summary of the combined modelling strategies to probe structural rearrangements in delithiated O2-Li_1.2–x_Mn_0.8_O_2_. At top of charge, ribbon-superstructure Li_0.2_Mn_0.8_O_2_ is predicted to be metastable by DFT structural relaxation but rearranges during experimental timescales. The kinetics and thermodynamics of these structural rearrangements can be modelled using AIMD and cluster expansion Monte Carlo, respectively. Modelling kinetic processes with AIMD and hybrid DFT relaxations identifies the O-redox mechanism as initiated by interlayer O–O dimerization, forming stable O_2_ molecules. Thermodynamic modelling using a cluster expansion model and Monte Carlo simulations identifies the formation of nanoscale voids containing O_2_. AIMD characterizes O_2_ in the nanovoids as a high-density nanoconfined fluid. Green, blue and orange circles show Li^+^, Mn and lattice O ions, respectively. Red circles show O atoms in O–O dimers. Blue shading shows MnO_6_ octahedra.

The structural rearrangements that occur during the cycling of Li-rich oxide cathodes introduce challenges for experimental characterization and atomistic modelling of their O-redox behaviour. The previous absence of suitable modelling strategies to account for both the kinetics and thermodynamics of these structural rearrangements^9^ has resulted in uncertainty about the mechanisms of O redox. Here, we have shown how combining AIMD and cluster expansion Monte Carlo simulations can resolve these structural changes over atomistic and nanoscale lengths while accounting for the kinetics and thermodynamics of this structural rearrangement (Fig. 5).

Our results show that O_2_ molecules can form in the bulk through kinetically accessible pathways. Peroxide and superoxide species form as transient reaction intermediates along these pathways and are not the final O-redox product at the top of charge. This result is consistent with high-resolution resonant inelastic X-ray scattering, neutron pair distribution function and superconducting quantum interference device magnetometry measurements of several lithium-rich cathodes, which provide evidence for molecular O_2_ confined in the bulk at the top of charge and the absence of peroxide or superoxide species^31,38,40–44^.

The convex hull of formation energies for Mn_*x*_O_2_ (Fig. 3a) shows that there is a thermodynamic driving force at the top of charge for Mn_0.8_O_2_ to phase segregate into MnO_2_ and molecular O_2_, whether this O_2_ is lost from the system or confined in the bulk. O_2_ loss and bulk O_2_ formation, therefore, can be considered to be different outcomes of the same thermodynamically driven process, where O^2–^ ions are oxidized to molecular O_2_ (ref. ^38^). O_2_ formed in the bulk is unstable with respect to gas-phase O_2_, and bulk O_2_ may subsequently escape through the surface of the cathode, thereby contributing to net O_2_ loss.

While O_2_ formed at the surface is expected to be lost immediately, as indicated by online electrochemical mass spectrometry data^32^, the degree to which O_2_ formed in the bulk will be lost depends on the ability of this O_2_ to diffuse through the bulk material and on the permeability of the cathode surface to O_2_ molecules. Our AIMD data indicate that O_2_ can diffuse through the bulk via a network of voids formed by the O-redox mechanism. When this void network percolates, as we predict it does for Mn_0.8_O_2_, long-range O_2_ diffusion from the cathode interior to the surface is feasible. Surface O_2_ loss during the first cycle, however, is understood to be accompanied by surface densification^32^, and these densified surfaces are expected to be relatively impermeable to O_2_, preventing the loss of bulk-formed O_2_ from the particle. This model of O redox is consistent with the previously proposed mechanism of O redox where O_2_ forms throughout the cathode, with O_2_ formed at the surface being lost while O_2_ in the bulk is trapped^38^. Any bulk-formed O_2_ that remains in the cathode is available to be reduced back to O^2–^ upon discharge, providing a mechanism for reversible O-redox capacity. Resonant inelastic X-ray scattering measurements of other Li-rich cathode materials show evidence for molecular O_2_ being present at the top of charge, but not after full discharge, which is consistent with the reduction of trapped bulk O_2_ back to lattice O^2−^ (refs. ^31,40^).

Our thermodynamic analysis indicates that in, Li_*x*_Mn_0.8_O_2_, bulk O_2_ formation and O_2_ loss can be considered to be parallel outcomes of the same thermodynamically favoured process. This link between bulk O_2_ formation and surface O_2_ loss probably extends to other cathode materials. If a material demonstrates oxygen loss as gas-phase O_2_ upon charging, this indicates a thermodynamic tendency of the parent phase to phase-separate to a mixture of an O-deficient phase and molecular O_2_. If the phase separation energy is sufficiently large, molecular O_2_ formation will be thermodynamically favoured irrespective of whether the resulting O_2_ is released as a gas or confined in the bulk. Experimental observations of O_2_ loss, widely observed in other Li-rich^12,45^ and conventional layered oxides^46,47^, may therefore be an indicator for materials that are thermodynamically unstable with respect to bulk O_2_ formation. Whether these materials do form bulk O_2_ will depend on the precise mechanisms of O redox in each case, which may potentially be resolved by applying the methods used in this study.

Our study also explains experimental observations of disordering and nanostructuring as being driven by O redox. Confined O_2_ in the bulk cannot easily be directly imaged. Nevertheless, the phase segregation in our Monte Carlo simulations reveals that Mn-deficient nanovoids are a signature of bulk O_2_ formation. This structural description is consistent with observations of nanovoids in cycled cathodes from X-ray and neutron pair distribution function, small-angle X-ray and neutron scattering and electron microscopy experiments^14–17,48^. Some studies have attributed the formation of voids to the presence of oxygen vacancies, often in the context of oxygen loss^15^. Our results indicate that nanovoids can form even without oxygen loss; oxygen atoms can instead dimerize to form molecular O_2_ species near the vacated O sites. Our results also indicate that these nanovoids can form throughout the Li_1.2–*x*_Mn_0.8_O_2_ material; it is not necessary for voids to form at the surface and then propagate into the bulk. We expect design strategies that stabilize the bulk and the surface against the formation of nanovoids to be important in preventing structural degradation and a loss of energy density in Li-rich cathodes.

Our large-scale models shine new light upon the properties of the confined O_2_ molecules. Previous studies using ^17^O NMR and AIMD have suggested that small numbers of O_2_ molecules are rigidly caged within their local environment^40,49^, with no long-range diffusion. Our results show a thermodynamic drive to form large voids that contain large numbers of O_2_ molecules. Within the voids, these O_2_ molecules are mobile and collectively can be considered as a high-density supercritical nanoconfined fluid. Individual O_2_ molecules in Mn vacancy clusters may have a more solid-like character, but as the Mn-deficient voids increase in size, we predict that O_2_ within these voids will increasingly display fluid-like behaviour.

The fluid character of O_2_ highlights important links between O_2_ formation in the bulk, the cathode nanostructure, O_2_ loss through the surface and voltage fade. Voltage fade has been associated with gradual O_2_ loss from the bulk in oxide cathodes such as the lithium-rich nickel–manganese–cobalt system at high states of charge through a mechanism of oxygen vacancy transport from bulk to surface^50^. Our results suggest an alternative oxygen transport mechanism in which nanoconfined fluid O_2_ diffuses through a percolating network of voids.

We calculate a local diffusion coefficient for nanoconfined O_2_ of ~10^–7^ cm^2^ s^–1^ in Li_0.2_Mn_0.8_O_2_. This calculated value is much higher than the macroscopic oxygen diffusion coefficient reported previously for Li-rich nickel–manganese–cobalt (~10^–17^ cm^2^ s^–1^)^50^ from experimental X-ray absorption and ptychography measurements. Long-range diffusion rates are likely to be limited by other factors such as residual lithium in the structure, the high tortuosity and slow formation kinetics of the void network and influenced by the variation in structure and composition of different Li-rich cathodes (Supplementary Note 3). Coatings and surface structures that prevent O_2_ release from the bulk of cathode materials are potentially important to preserve long-term cycling stability. However, these alone will not be able to inhibit structural rearrangements in the bulk, where most of the O_2_ is formed. Designing bulk structures with void networks that do not interconnect is also a possible strategy for inhibiting bulk oxygen transport, thereby minimizing oxygen loss from the surface.

In conclusion, the combination of AIMD and cluster expansion-based Monte Carlo simulation allows the detailed atomic-scale exploration of a lithium-rich O-redox cathode by predicting realistic structures of the charged cathode material. Using this approach, we have identified a thermodynamically favourable and kinetically viable O-redox mechanism to form nanoconfined O_2_ molecules in the bulk structure with Mn migration. Long-term cycling of the cathode results in local phase segregation into MnO_2_-rich regions and Mn-deficient nanovoids that contain O_2_ molecules as a nanoconfined fluid. These nanovoids are connected in a percolating network that can facilitate oxygen diffusion, which provides a mechanistic link between O_2_ formation in the bulk and O_2_ loss through the surface. The combined methodology presented here answers long-standing questions about the atomic- to nanoscale mechanisms of O-redox in Li-rich Mn-based cathodes and highlights directions for improving the performance of other high-energy density cathodes that display structural rearrangements during cycling.

## Methods

### AIMD with GGA + *U* DFT

Generalized gradient approximation (GGA) + *U* AIMD simulations were run with the VASP code^51–55^. Electronic exchange and correlation were approximated using the Perdew–Burke–Ernzerhof + *U* functional. A Hubbard *U* parameter of 3.9 eV was applied to the Mn 3*d* orbitals to correct for the self-interaction error. Dispersion corrections were included through Grimme’s semiclassical D3 scheme^56–58^. All AIMD calculations used a plane-wave cutoff of 400 eV, a single Γ point for **k**-space sampling and a time step of 2 fs. The lattice parameters were kept fixed to the zero-pressure 0% optimized values obtained from hybrid DFT geometry relaxations.

To sample structural transformations pathways in Li_0.2_Mn_0.8_O_2_ (Fig. 2), simulations were performed at 300, 600 and 900 K, starting from the delithiated framework (Supplementary Fig. 2), using a (1 × 2 × 1) expansion of the crystallographic unit cell of the ribbon superstructure (160 atoms). For each AIMD simulation, two equilibration stages were performed: first, a 2 ps run in the microcanonical (NVE) ensemble (with constant composition, volume and energy) by ramping the temperature up from 0 K to the target temperature with temperature rescaling every 50 steps; and second, a 2 ps run in the canonical (NVT) ensemble (with constant composition, volume and temperature) at the target temperature. The equilibration steps were designed to smooth any energy and temperature fluctuations in the system. Following the equilibration, we ran production simulations from which we sampled structures along the trajectories by assessing the formation or breaking of short (<1.7 Å, <1.4 A or <1.3 Å) O–O bonds or the movement of Mn ions out of the TM layer; if either of these events occurred (for example, a peroxide bond (1.4 Å < *d*_O–O_ < 1.7 Å) formed from two neighbouring O atoms bonded to Mn or a molecular O_2_ bond (*d*_O–O_ < 1.3 Å) formed from a superoxide species (1.3 Å < *d*_O–O_ < 1.4 Å)), the structures were extracted from the AIMD simulations and fully relaxed with hybrid DFT calculations. GGA functionals over-estimate O–O bond lengths, so the demarcation points for these different O–O species bond lengths are slightly longer than used previously^25^.

To fit the cluster expansion, AIMD was initially used to sample different, low-energy O–O configurations for each cation arrangement (as discussed in Supplementary Note 1). These sampling simulations were run at 300 K, in the NVT ensemble with velocities initialized to replicate the Boltzmann distribution. Structures along the trajectory displaying newly formed O–O bonds were extracted and fully relaxed with hybrid DFT.

AIMD was also used to examine the mobility of O–O dimers in low-energy structures obtained from the cluster expansion (Fig. 4). These simulations were run at 300 K in the NVT ensemble, with the same 2 ps NVE and 2 ps NVT equilibration procedure as used for the structural transformation sampling simulations. The simulations were run for 60 ps.

AIMD was used to compute the RDF of liquid O_2_. These simulations were run in a cubic box containing 256 O atoms (128 O_2_ molecules), with an *a* lattice parameter of 18.22202 Å (equivalent to the density of liquid O_2_ at *T* = 88 K, 1.141 g cm^–3^). These simulations were run at 300 K, in the NVT ensemble (with fixed cell volume and lattice parameters). The system was equilibrated with a 2 ps NVE, followed by a 2 ps NVT equilibration step. The RDF was calculated from a 10 ps trajectory.

### Hybrid DFT geometry relaxations

Hybrid-exchange DFT geometry relaxations were performed to obtain high-accuracy results for selected structures along the GGA + *U* AIMD structural transformation trajectory and for the set of structures and energies to train the cluster expansion. Hybrid DFT relaxations were performed using the CRYSTAL17 code, which uses local basis sets^59^. Electronic exchange and correlation were approximated using the screened hybrid-exchange functional HSE06^60,61^, with dispersion forces included using Grimme’s semiclassical D3 scheme^56–58^. All-electron atom-centred Gaussian basis sets were used for all atoms, available from the CRYSTAL online database (www.crystal.unito.it), with the online labels: Li (Li_5- 11(1d)G_baranek_2013_LiNbO3), Mn (Mn_86-411d41G_towler_1992) and O (O_8-411d1_cora_2005). The Coulomb overlap and penetration, exchange overlap, and g- and n-series exchange penetration were truncated with thresholds of 10^–7^, 10^–7^, 10^–7^, 10^–7^ and 10^–14^, respectively, as described in the CRYSTAL documentation. Reciprocal space was sampled using a 2 × 6 × 4 Γ-centred Monkhorst–Pack mesh^62^ for the ~80 atom (1 × 1 × 1) crystallographic unit cell of ribbon-superstructure Li_*x*_Mn_0.8_O_2_, and a 2 × 3 × 4 mesh for the ~160 atom (1 × 2 × 1) supercell. The self-consistent field procedure was performed up to a convergence threshold of *∆E* = 10^–7^ Hartree per unit cell. Calculations were initialized with Mn ions in a ferromagnetic alignment. Full geometry optimizations of lattice parameters and atomic positions, in the absence of any symmetry constraints, were performed using the default convergence criteria in CRYSTAL17.

### Cluster expansion

A cluster expansion model of Li_0_Mn_0.8_O_2_ was trained using ICET code^63^. The cluster expansion implemented a binary Mn vacancy basis, using the eight-atom primitive cell of an O2-layered LiTMO_2_ structure. The cluster expansion was fitted with energies and structures from 632 hybrid DFT calculations in cells containing either 80 or 160 sites (including cation vacancies). The training data included compositions along the O_2_–MnO_2_–MnO tie line to ensure that the local phase-segregation behaviour of Mn_0.8_O_2_ was accurately captured. Relaxed structures from the hybrid DFT calculations were mapped back onto the primitive unit cell lattice using the mapping tools in the ICET code. As discussed in Supplementary Note 1, oxygen atoms were mapped first to their nearest lattice sites. Cations were mapped subsequently, similar to a strategy reported previously for structures that exhibit large relaxations^64^. The fit consisted of pair interactions up to 7.5 Å, triplet interactions up to 4.5 Å and quadruplet interactions up to 4.5 Å with the sum truncated at this point. The effective cluster interactions (ECIs) were obtained using the automatic relevance determination regression algorithm (ARDR), with a recursive feature elimination (RFE) approach, in which minimally contributing parameters are removed recursively and a cross-validation score calculated, repeated until the cross-validation score no longer improves. The training set structures were generated in three ways. An initial set was generated at random, based upon enumerating Li–Mn vacancy orderings within (1 × 1 × 1) and (1 × 2 × 1) expansions of the conventional unit cell of the delithiated ribbon-structured material (Supplementary Fig. 1b). A second set of ‘targeted’ structures containing O–O configurations were generated, which were established to be low energy (Supplementary Note 1 and Supplementary Figs. 4–6). Using this set of random and targeted structures, an initial, low-accuracy cluster expansion was trained. The parameterized cluster expansion Hamiltonian was then used to run lattice Monte Carlo annealing simulations in (1 × 1 × 1) and (1 × 2 × 1) expansions of the conventional cell of Li_*x*_Mn_0.8_O_2_, with compositions of Li_0_Mn_*x*_O (0 ≤ *x* ≤ 1), to generate additional structures that were relaxed and added to re-train the cluster expansion. This procedure of training, running MC, sampling new structures and re-training was performed several times to improve the quality of the fit. The final, fully-trained cluster expansion with the ARDR + RFE approach was fit with 60 non-zero ECIs and a *k*-fold cross-validation root-mean-squared error of 184 meV per primitive cell (equivalent to 23 meV per atom). Details of the fit are shown in Supplementary Figs. 8 and 9.

### Lattice Monte Carlo

Lattice Monte Carlo simulations were performed using the ‘mchammer’ module within ICET. Using the parameterized cluster expansion, the internal energy of Li_0_Mn_0.8_O_2_ was calculated as a function of temperature from a set of canonical ensemble Monte Carlo simulations using the Metropolis–Hastings algorithm. The simulations were run in the canonical annealing mode to obtain low-energy structures. Simulations started at a temperature of 300 K and reached a temperature of 0 K. In our workflow, we used Monte Carlo for two purposes. Firstly, as described above, to re-train the cluster expansion, we generated new structures from Monte Carlo annealing in (1 × 1 × 1) and (1 × 2 ×1) expansions of the conventional crystallographic unit cell of Li_*x*_Mn_0.8_O_2_. These simulations were run for 250,000 steps. Secondly, for the production annealing simulations (Fig. 3) we used a (5 × 15 × 8) supercell expansion of the conventional unit cell, which contained 48,000 sites, where sites are Mn, O and *X* (where *X* is a cation vacancy). These simulations were run for 1,000,000 steps. We checked for size consistency by conducting equivalent Monte Carlo runs in supercells with (2 × 6 × 3) and (3 × 9 × 5) expansions of the conventional unit cell of Li_*x*_Mn_0.8_O_2_, containing 2,880 and 10,800 sites, respectively, and evaluating the O environments and percolating properties of the O_2_ network.

## Online content

Any methods, additional references, Nature Portfolio reporting summaries, source data, extended data, supplementary information, acknowledgements, peer review information; details of author contributions and competing interests; and statements of data and code availability are available at 10.1038/s41563-024-01873-5.

### Supplementary information

Supplementary Information Supplementary Notes 1-4, Table 1 and Figs. 1–14.

## Data Availability

A complete dataset for the computational modelling and analysis described in this paper is available at *Zenodo* (10.5281/zenodo.11068469) (ref. ^65^) and will be available from the University of Bath Research Data Archive. This dataset contains inputs and outputs for all DFT calculations plus scripts for analysis of the DFT data and for plotting Figs. 2–4. A subsidiary dataset containing only the figure-plotting scripts and relevant input data is available on GitHub (https://github.com/kitmccoll/data-phase_segregation_nanoconfined_fluid_O2).

## Code availability

Analysis of the results used the ASE^66^ and Pymatgen^67^ Python libraries, as well as NumPy^68^, SciPy^69^ and Matplotlib^70^. The prevalence of different polyhedral in the final structure from the Monte Carlo simulations was analysed using the polyhedral_analysis code^71^. Diffusion was analysed using the kinisi code^72,73^. Structures were visualized using VESTA^74^.

## References

[1] Koga H (2013). Reversible oxygen participation to the redox processes revealed for Li_1.20_Mn_0.54_Co_0.13_Ni_0.13_O_2_. J. Electrochem. Soc..

[2] Sathiya M (2013). Reversible anionic redox chemistry in high-capacity layered-oxide electrodes. Nat. Mater..

[3] Croy, J. R. et al. Examining hysteresis in composite *x*Li_2_MnO_3_⋅(1–*x*)LiMO_2_ cathode structures. *J. Phys. Chem. C* **117**, 6525–6536 (2013).

[4] Gallagher KG (2013). Correlating hysteresis and voltage fade in lithium- and manganese-rich layered transition-metal oxide electrodes. Electrochem. Commun..

[5] Li B (2021). Correlating ligand-to-metal charge transfer with voltage hysteresis in a Li-rich rock-salt compound exhibiting anionic redox. Nat. Chem..

[6] Gent WE (2017). Coupling between oxygen redox and cation migration explains unusual electrochemistry in lithium-rich layered oxides. Nat. Commun..

[7] Hong J (2019). Metal–oxygen decoordination stabilizes anion redox in Li-rich oxides. Nat. Mater..

[8] Gent WE, Abate II, Yang W, Nazar LF, Chueh WC (2020). Design rules for high-valent redox in intercalation electrodes. Joule.

[9] Zhang M (2022). Pushing the limit of 3*d* transition metal-based layered oxides that use both cation and anion redox for energy storage. Nat. Rev. Mater..

[10] Van der Ven A, Deng Z, Banerjee S, Ong SP (2020). Rechargeable alkali-ion battery materials: theory and computation. Chem. Rev..

[11] Tygesen AS, Chang JH, Vegge T, García-Lastra JM (2020). Computational framework for a systematic investigation of anionic redox process in Li-rich compounds. NPJ Comput. Mater..

[12] Armstrong AR (2006). Demonstrating oxygen loss and associated structural reorganization in the lithium battery cathode Li[Ni_0.2_Li_0.2_Mn_0.6_]O_2_. J. Am. Chem. Soc..

[13] Zheng J (2015). Structural and chemical evolution of Li- and Mn-rich layered cathode material. Chem. Mater..

[14] Hu E (2018). Evolution of redox couples in Li- and Mn-rich cathode materials and mitigation of voltage fade by reducing oxygen release. Nat. Energy.

[15] Yan P (2019). Injection of oxygen vacancies in the bulk lattice of layered cathodes. Nat. Nanotechnol..

[16] Grenier A (2021). Nanostructure transformation as a signature of oxygen redox in Li-rich 3*d* and 4*d* cathodes. J. Am. Chem. Soc..

[17] Zhao E (2022). Quantifying the anomalous local and nanostructure evolutions induced by lattice oxygen redox in lithium-rich cathodes. Small Methods.

[18] Ben Yahia M, Vergnet J, Saubanère M, Doublet M-L (2019). Unified picture of anionic redox in Li/Na-ion batteries. Nat. Mater..

[19] Seo D-H (2016). The structural and chemical origin of the oxygen redox activity in layered and cation-disordered Li-excess cathode materials. Nat. Chem..

[20] Okubo M, Yamada A (2017). Molecular orbital principles of oxygen-redox battery electrodes. ACS Appl. Mater. Interfaces.

[21] Radin MD, Vinckeviciute J, Seshadri R, Van der Ven A (2019). Manganese oxidation as the origin of the anomalous capacity of Mn-containing Li-excess cathode materials. Nat. Energy.

[22] Chen H, Islam MS (2016). Lithium extraction mechanism in Li-rich Li_2_MnO_3_ involving oxygen hole formation and dimerization. Chem. Mater..

[23] Lee E, Persson KA (2014). Structural and chemical evolution of the layered Li-excess Li_*x*_MnO_3_ as a function of Li content from first-principles calculations. Adv. Energy Mater..

[24] Kim B (2022). A theoretical framework for oxygen redox chemistry for sustainable batteries. Nat. Sustain..

[25] Vinckeviciute J, Kitchaev DA, Van der Ven A (2021). A two-step oxidation mechanism controlled by Mn migration explains the first-cycle activation behavior of Li_2_MnO_3_-based Li-excess materials. Chem. Mater..

[26] Kitchaev DA, Vinckeviciute J, Van der Ven A (2021). Delocalized metal–oxygen π-redox is the origin of anomalous nonhysteretic capacity in Li-ion and Na-ion cathode materials. J. Am. Chem. Soc..

[27] Saubanère M, McCalla E, Tarascon J-M, Doublet M-L (2016). The intriguing question of anionic redox in high- energy density cathodes for Li-ion batteries. Energy Environ. Sci..

[28] Delmas C, Fouassier C, Hagenmuller P (1980). Structural classification and properties of the layered oxides. Phys. BC.

[29] Eum D (2020). Voltage decay and redox asymmetry mitigation by reversible cation migration in lithium-rich layered oxide electrodes. Nat. Mater..

[30] Cao X (2021). Achieving stable anionic redox chemistry in Li-excess O2-type layered oxide cathode via chemical ion- exchange strategy. Energy Storage Mater..

[31] House RA (2020). Superstructure control of first-cycle voltage hysteresis in oxygen-redox cathodes. Nature.

[32] Luo K (2016). Charge-compensation in 3*d*-transition-metal-oxide intercalation cathodes through the generation of localized electron holes on oxygen. Nat. Chem..

[33] Eum D (2022). Coupling structural evolution and oxygen-redox electrochemistry in layered transition metal oxides. Nat. Mater..

[34] House RA (2023). Delocalized electron holes on oxygen in a battery cathode. Nat. Energy.

[35] Benedek R (2018). First-cycle simulation for Li-rich layered oxide cathode material *x*Li_2_MnO_3_⋅(1–*x*)LiMO_2_ (*x* = 0.4). J. Electrochem. Soc..

[36] Zhang Z, Zhao S, Wang B, Yu H (2020). Local redox reaction of high valence manganese in Li_2_MnO_3_-based lithium battery cathodes. Cell Rep. Phys. Sci..

[37] Liu T (2022). Origin of structural degradation in Li-rich layered oxide cathode. Nature.

[38] House RA (2021). The role of O_2_ in O-redox cathodes for Li-ion batteries. Nat. Energy.

[39] Freiman YuA, Jodl HJ (2004). Solid oxygen. Phys. Rep..

[40] House RA (2020). First-cycle voltage hysteresis in Li-rich 3*d* cathodes associated with molecular O_2_ trapped in the bulk. Nat. Energy.

[41] House RA (2021). Covalency does not suppress O_2_ formation in 4*d* and 5*d* Li-rich O-redox cathodes. Nat. Commun..

[42] House RA (2022). Detection of trapped molecular O_2_ in a charged Li-rich cathode by neutron PDF. Energy Environ. Sci..

[43] McColl K (2022). Transition metal migration and O_2_ formation underpin voltage hysteresis in oxygen-redox disordered rocksalt cathodes. Nat. Commun..

[44] Boivin E (2021). Bulk O_2_ formation and Mg displacement explain O-redox in Na_0.67_Mn_0.72_Mg_0.28_O_2_. Joule.

[45] Lee J (2017). Mitigating oxygen loss to improve the cycling performance of high capacity cation-disordered cathode materials. Nat. Commun..

[46] Jung R, Metzger M, Maglia F, Stinner C, Gasteiger HA (2017). Oxygen release and its effect on the cycling stability of LiNi_*x*_Mn_*y*_Co_*z*_O_2_ (NMC) cathode materials for Li-ion batteries. J. Electrochem. Soc..

[47] Sharifi-Asl S, Lu J, Amine K, Shahbazian-Yassar R (2019). Oxygen release degradation in Li-ion battery cathode materials: mechanisms and mitigating approaches. Adv. Energy Mater..

[48] Sun C (2020). High-voltage cycling induced thermal vulnerability in LiCoO_2_ cathode: cation loss and oxygen release driven by oxygen vacancy migration. ACS Nano.

[49] Sharpe R (2020). Redox chemistry and the role of trapped molecular O_2_ in Li-rich disordered rocksalt oxyfluoride cathodes. J. Am. Chem. Soc..

[50] Csernica PM (2021). Persistent and partially mobile oxygen vacancies in Li-rich layered oxides. Nat. Energy.

[51] Kresse G, Furthmüller J (1996). Efficiency of ab-initio total energy calculations for metals and semiconductors using a plane-wave basis set. Comput. Mater. Sci..

[52] Kresse G, Hafner J (1993). Ab initio molecular dynamics for liquid metals. Phys. Rev. B.

[53] Kresse G, Hafner J (1994). Ab initio molecular-dynamics simulation of the liquid-metal–amorphous-semiconductor transition in germanium. Phys. Rev. B.

[54] Kresse G, Joubert D (1999). From ultrasoft pseudopotentials to the projector augmented-wave method. Phys. Rev. B.

[55] Kresse G, Furthmüller J (1996). Efficient iterative schemes for ab initio total-energy calculations using a plane-wave basis set. Phys. Rev. B.

[56] Grimme S, Antony J, Ehrlich S, Krieg H (2010). A consistent and accurate ab initio parametrization of density functional dispersion correction (DFT-D) for the 94 elements H–Pu. J. Chem. Phys..

[57] Grimme S, Ehrlich S, Goerigk L (2011). Effect of the damping function in dispersion corrected density functional theory. J. Comput. Chem..

[58] Grimme S, Hansen A, Brandenburg JG, Bannwarth C (2016). Dispersion-corrected mean-field electronic structure methods. Chem. Rev..

[59] Dovesi R (2018). Quantum-mechanical condensed matter simulations with CRYSTAL. WIREs Comput. Mol. Sci..

[60] Heyd J, Scuseria GE, Ernzerhof M (2003). Hybrid functionals based on a screened Coulomb potential. J. Chem. Phys..

[61] Heyd J, Scuseria GE (2004). Efficient hybrid density functional calculations in solids: assessment of the Heyd–Scuseria–Ernzerhof screened Coulomb hybrid functional. J. Chem. Phys..

[62] Monkhorst HJ, Pack JD (1976). Special points for Brillouin-zone integrations. Phys. Rev. B.

[63] Ångqvist M (2019). ICET – a Python library for constructing and sampling alloy cluster expansions. Adv. Theory Simul..

[64] Yang JH, Chen T, Barroso-Luque L, Jadidi Z, Ceder G (2022). Approaches for handling high-dimensional cluster expansions of ionic systems. NPJ Comput. Mater..

[65] McColl, K. kitmccoll/data-phase_segregation_nanoconfined_fluid_O2: v1.0.0. *Zenodo*10.5281/zenodo.11068469 (2024).

[66] Larsen AH (2017). The atomic simulation environment—a Python library for working with atoms. J. Phys. Condens. Matter.

[67] Ong SP (2013). Python Materials Genomics (pymatgen): a robust, open-source python library for materials analysis. Comput. Mater. Sci..

[68] Harris CR (2020). Array programming with NumPy. Nature.

[69] Virtanen P (2020). SciPy 1.0: fundamental algorithms for scientific computing in Python. Nat. Methods.

[70] Hunter JD (2007). Matplotlib: a 2D graphics environment. Comput. Sci. Eng..

[71] Morgan, B. J. Polyhedral analysis. *GitHub* https://github.com/bjmorgan/polyhedral-analysis (2020).

[72] McCluskey AR, Squires AG, Dunn J, Coles SW, Morgan BJ (2024). kinisi: Bayesian analysis of mass transport from molecular dynamics simulations. J. Open Source Softw..

[73] McCluskey, A. R., Coles, S. W. & Morgan, B. J. Accurate estimation of diffusion coefficients and their uncertainties from computer simulation. Preprint at 10.48550/arXiv.2305.18244 (2023). 10.1021/acs.jctc.4c01249 PMC11736684 39807535

[74] Momma K, Izumi F (2011). VESTA3 for three-dimensional visualization of crystal, volumetric and morphology data. J. Appl. Crystallogr..

