# Supplementary material for: Phase segregation and nanoconfined fluid O2 in a lithium-rich oxide cathode
Source: Nat Mater. 2024 May 13;23(6):826–33. doi: 10.1038/s41563-024-01873-5 (PMC11150156; doi:10.1038/s41563-024-01873-5)
Supplement: Supplementary file 1 — Supplementary Notes 1-4, Table 1 and Figs. 1–14. [file 41563_2024_1873_MOESM1_ESM.pdf]

nature materials

Article

https://doi.org/10.1038/s41563-024-01873-5

Phase segregation and nanoconfined fluid O_2 in a lithium-rich oxide cathode

In the format provided by the authors and unedited

Contents

Supplementary Notes

1. Oxygen redox cluster expansion
2. Training the cluster expansion with a confined O_2 endmember
3. Comparison of host-framework structural rearrangements obtained from AIMD simulations and cluster expansion
4. Li-rich cathodes with different structures: O2 vs. O3 stacking

Supplementary Results

Supplementary Table 1

Supplementary Figures 1 - 14

References

Supplementary Notes

Supplementary Note 1. Oxygen redox cluster expansion.

Modelling an oxygen redox cathode material using a cluster expansion (CE) involves addressing complexities that are not typically present in CEs used to model traditional cathodes, such as $LiCoO_2$. Most importantly, a CE for O-redox must be able to describe O_2 molecules using a lattice model, despite the fact that O atoms may have moved far off-lattice to form O_2. A complete overview of the CE methodology is described in detail in several reviews;[1–3] here we discuss only these features relevant for modelling O-redox cathode materials.

The CE relies on a lattice model, and fitting a successful CE requires that: i) atoms in relaxed structures can be mapped back to sites on a lattice and ii) each lattice configuration corresponds to a unique relaxed structure and its energy.[3] There are two key characteristics of the $Mn_{0.8}O_2$ system that mean a CE can successfully be fitted. Firstly, O_2 molecules always correspond to specific sites in the lattice model: O–MnX_5 and O–X_6 (where 'X' is an explicitly defined vacancy cation site). For a given cation arrangement, the lowest energy relaxed structure is always when O atoms, initially in O–MnX_5 and O–X_6 sites in the lattice, relax off-lattice to form O_2 molecules (as opposed to O atoms in O–MnX_5 in lattice sites remaining bonded only to Mn after relaxation). Secondly, the fluid character of O_2 molecules at room temperature (Figure 4, Main Text), means that the rotational and translational arrangements of the O_2 can be considered equivalent, and O atoms in O_2 can be mapped to any nearby site. We elaborate upon these features below.

Firstly, for a given cation configuration, (*i.e.*, for each lattice structure) a single relaxed structure and energy is required to train the CE. Yet, there are multiple possible relaxed structures, corresponding to different numbers of O_2 molecules, and some of these relaxed structures are metastable (Supplementary Figure 5). For example, oxygen atoms in lattice O–MnX_5 sites, when relaxed, could either remain bonded to only Mn, or could be displaced off-lattice to form O_2 molecules with O atoms originating from other O–MnX_5 sites, or O–X_6 sites. We note that O–X_6 sites in a lattice starting configuration have a barrierless mechanism to dimerise with other O atoms (Supplementary Figure 5a–d), so there are no metastable relaxed structures where O–X_6 sites do not form O_2. To determine which relaxed configurations are the lowest energy, we assessed many possible stable and metastable structures which map to the same lattice (i.e., which have the same cation configuration). These structures contained O atoms either i) bonded to a single Mn, or ii) forming O_2 molecules. We found that the minimum energy structure is always when O atoms in O–MnX_5 (and O–X_6) lattice sites form O_2 molecules (Supplementary Figure 5). There is a ~2.5 eV drop in energy for every pair of O atoms bonded to only Mn that dimerise to form O_2 (Supplementary Figure 5e). Similarly, taking a structure containing several O_2 molecules, and manually and sequentially breaking the O–O bonds to re-combine the O with undercoordinated Mn, there is a ~2.5 eV increase in energy each time an O_2 molecule dissociates (Supplementary Figure 6). Thus, for a given cation configuration (*i.e.,* starting from any lattice configuration), the lowest energy relaxed structure is always when the number of O_2 molecules from O–MnX_5 and O–X_6 sites is maximised. For the purposes of fitting the cluster expansion, before structures were mapped and added to training set, a check was performed on each relaxed structure to ensure that it did not contain any O atoms bonded to a single Mn. Any structures with this feature (which are metastable) were either i) discarded and not added to the training set, or ii) all O atoms in those sites were forced to dimerise with neighbouring O atoms with only one Mn neighbour, and the structure re-relaxed before being added to the training set. This means that the cluster expansion was always trained on the lowest energy relaxed structure for that given lattice configuration. This also ensures that in the lattice model (including in lattice structures generated by the Monte Carlo simulations), O–MnX_5 and O–X_6 sites always represent O_2 molecules.

Secondly, having established that the lowest energy structures always have a maximum number of O_2 molecules, these O_2 molecules may rotate and translate. Two questions arise. i) Is it possible (or even

necessary) for the cluster expansion Hamiltonian to capture the rotations and translations? ii) Can O_2 molecules that have translated far off-lattice be mapped successfully? Both issues are resolved by the observation, from ab initio molecular dynamics simulations, that O_2 molecules have a fluid character at room temperature (~300K) (Figure 4, Main Text). The O_2 molecules freely rotate and translate to access all the space within the voids and can move through the voids when the voids connect and percolate.

Resulting from the room-temperature O_2 mobility, it is not realistic for the cluster expansion Hamiltonian to capture the O_2 rotations and translations. Furthermore, is it not important from an energetic perspective. To determine the energetics of the rotations and translations, we ran a 100ps AIMD simulation on the structure in Supplementary Figure 5d, took 20 snapshots of different local arrangements of the O_2 molecules (without any change to the cation arrangement), and fully relaxed the structures. The maximum range of energies spans ±3 meV atom^{-1} (Supplementary Figure 7, S8), which is below the error in the cluster expansion. This energy change is negligible compared the energy change with the breaking and formation O–O bonds, and that it is therefore not important for the CE to capture the rotation and translation of the O_2 molecules, meaning that all arrangements of the O_2 molecules could be approximated to be structurally and energetically equivalent. Given several relaxed structures, arising from the same lattice configuration, and containing the maximum possible number of O_2 molecules for that lattice configuration, we simply took the lowest energy as the value with which to train the cluster expansion. Regarding mapping, because the O_2 molecules can freely move through the structure, each O atom in an O_2 molecule could have originated from any O site within the void, and it is sufficient to map them to any O site. The O atoms can simply be mapped to their nearest site. As stated in the Methods, this mapping of O atoms was performed before mapping the cations.

As a result of this mapping strategy, and because O_2 molecules are always represented by specific local configurations (O–MnX_5 and O–X_6 sites), rather than separate species, there is no disorder on the O sublattice, meaning it is not explicitly included in the cluster expansion (Supplementary Figure 9). The cluster expansion basis is therefore Mn-vacancy (‘*X*’) only, and the representation of O_2 molecules emerges implicitly from specific Mn-vacancy configurations. This reflects the fact that in the real system, O_2 molecules form where there are sufficient Mn vacancies (Mn-vacancy clusters and Mn-deficient voids) to leave O atoms heavily undercoordinated (one or no Mn neighbours).

To efficiently obtain a good starting approximation for the lowest energy structure, given a lattice configuration, we wrote a piece of structure-manipulation code that would automatically dimerise all possible O–MnX_5 and O–X_6 lattice sites to form O_2 molecules. This pre-dimerised structure would then be fully relaxed (Supplementary Figure 10). To ensure that the CE is always fitted with the ground-state energy for each cation configuration, we applied a check to each mapped structure containing O–MnX_5 and O–X_6 sites, to establish whether, in the corresponding relaxed structure, those O sites formed O_2 molecules. If they did not (i.e., if any O atoms remained bonded to only a single Mn), we applied the code to the structure to dimerise those O sites and the structure was re-relaxed, or the structure was discarded.

Supplementary Note 2. Training the cluster expansion with a confined O_2 endmember

The cluster expansion (Figure 3a, main text) was fitted to represent O_2 molecules constrained in the bulk of the cathode. To obtain the internal energy of this structure, we used the crystallographic unit cell of $Li_{1.2}Mn_{0.8}O_2$ with a volume per O atom equivalent to the volume per O atom of ribbon-structures $Li_{0.2}Mn_{0.8}O_2$ at the top of charge, and removed all the Li and Mn species. The O atoms were the randomly displaced up to 0.2 Å from their lattice sites and were dimerised with their nearest neighbours to form 20 O_2 molecules. A short (20 ps) AIMD simulation was run at 300K in the NVT ensemble (*i.e.*, at a constant cell volume) using GGA+*U* (settings described above) to introduce orientational disorder into the arrangement of the O_2 molecules. From the AIMD trajectory, several images were extracted, and the atomic positions were relaxed with hybrid DFT whilst the

volume of the cell was kept constant. The energy of the most stable structure was used as the energy for confined O_2. To calculate the energy of O_2 gas (Figure 3a, main text), we relaxed an isolated O_2 molecule using hybrid DFT, and added the rotational and translational enthalpy, and experimentally measured entropy of O_2 at 1 atmosphere and 298 K.[4,5]

Supplementary Note 3. Comparison of host-framework structural rearrangements obtained from AIMD simulations and cluster expansion.

To highlight the effectiveness of the cluster expansion at obtaining low-energy structures, we used the CE to run 25 Monte Carlo annealing simulations of $Mn_{0.8}O_2$ in unit cells of 80-160 atoms. We then applied the dimerisation code to obtain an approximate low-energy starting structure, and then fully relaxed all the structures. The energy of these relaxed structures (Supplementary Figure 13) are plotted against the energy of all the structures used to train the cluster expansion, and the relaxed structures from the AIMD trajectory in Figure 2, Main Text (with remaining lithium extracted to match the composition $Mn_{0.8}O_2$). The pristine ribbon-superstructure framework is used as the reference energy (0 eV) and is the highest energy structure. The CE + MC annealed structures are the lowest energy and are far below those from the AIMD simulations. The lowest energy structure from the CE + MC annealing is ~35 eV/cell (~290 meV/atom) below the pristine structure. This enormous drop in energy highlights how far from equilibrium the pristine ribbon structure is and highlights the very large stabilising effect of the O2 formation and Mn rearrangement exhibited by the annealed structures. These results are conclusive evidence that the CE is an effective tool for obtaining low energy structures. As an additional demonstration of the predictive power of the CE, we have included details of the lowest energy structure that is predicted by the cluster expansion (Supplementary Figure 14). Following dimerisation and DFT relaxations, the structure features four O_2 molecules, clustered together, and most Mn ions in coordination similar to a MnO_2-like phase. The unit cell is too small to allow the system to 'truly' phase-segregate. Nevertheless, the structural features (O_2 clustering and MnO_2 like regions) of the small cell mirror the phase-segregation in larger cells.

Supplementary Note 4. Li-rich cathodes with different structures: O2 vs. O3 stacking

Our cluster expansion and lattice Monte Carlo simulations are based upon $Li_xMn_{0.8}O_2$ with an O2-layered structure, rather than the more common O3 layered structure. Nevertheless, some aspects of the results for O2–$Li_xMn_{0.8}O_2$ are likely to be applicable to an O3–$Li_xMn_{0.8}O_2$ structure. The convex hull (Figure 3a, main text) for the O_2–MnO_2–MnO tie line is calculated for the O2-layered system. An equivalent convex hull for an O3-system is likely to share the same characteristics. The position of the confined O_2 endmember will remain relatively unchanged in the O3 system, because the energy of confined O_2 depends on the volume per atom, whereas the effect of the stacking sequence will be negligible. The most-stable MnO_2 phase is likely to fall at similar or lower points on the plot, because λ–MnO_2 and δ–MnO_2 (which have an O3 oxygen sublattice) and known to be low-energy MnO_2 polymorphs.[4] It is unlikely that the stacking sequence will considerably affect the energies of the $Mn_{0.8}O_2$ structures, meaning that for the O3 system, these $Mn_{0.8}O_2$ compositions are likely to lie well-above the ground-state hull between O_2 and the most-stable O3-layered MnO_2 phase. We therefore predict that an O3–$Li_0Mn_{0.8}O_2$ will also be susceptible to phase-segregation and the formation of nanovoids containing O_2. We expect, however, that the nanovoid network will form with a distinctly different structure and perhaps different connectivity (i.e., different percolating properties) in the O2 and O3 systems, and this may result in different electrochemical properties between the two systems.

Supplementary Results

Supplementary Tables

Supplementary Table 1. Energies and structural properties of hybrid-DFT relaxed structures, sampled from the GGA+*U* AIMD trajectory at 900K (corresponding to structures I to IV in Figure 2, Main Text).

Structure	Relative energy (eV cell^{-1})	*a* (Å)	Δa (%)	*b* (Å)	Δb (%)	*c* (Å)	Δc (%)	Volume (Å^3)	ΔVolume (%)
I	0.00	14.14	0.0	9.84	0.0	8.94	0.0	1244.1	0.0
II	-1.59	14.26	0.9	9.80	-0.4	8.73	-2.4	1219.6	-2.0
III	-3.71	14.25	0.8	9.73	-1.1	9.43	5.5	1280.1	2.9
IV	-11.27	14.08	-0.4	9.80	-0.4	9.10	1.8	1255.4	0.9

Supplementary Figures

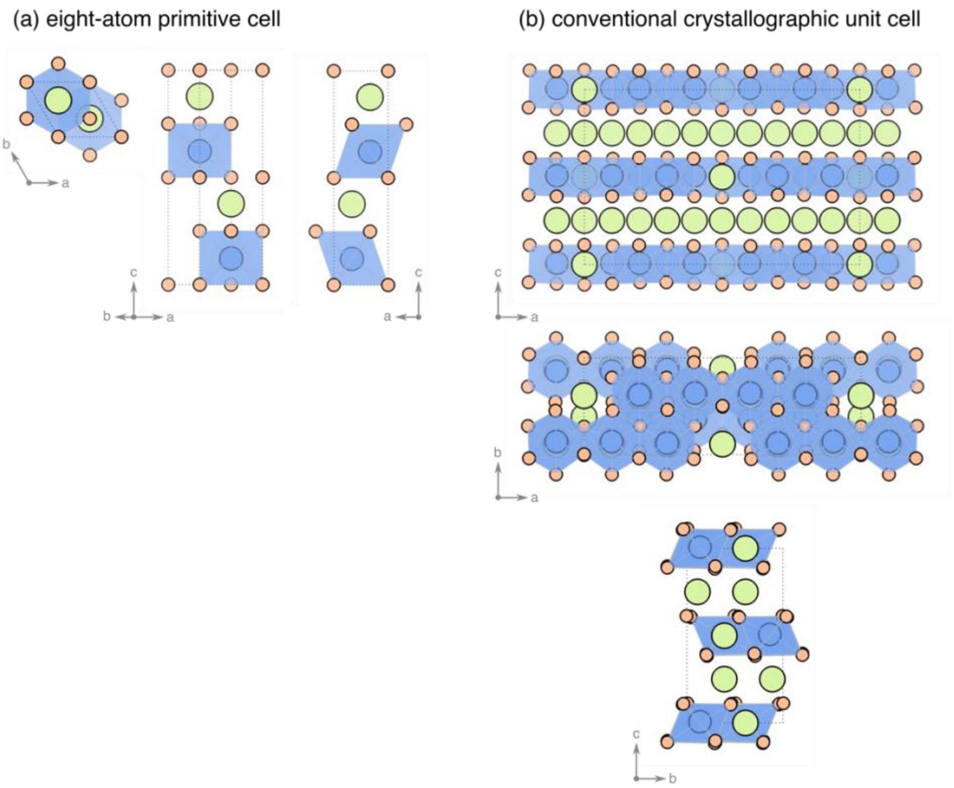

Supplementary Figure 1. (a) Hexagonal eight-atom primitive cell of an O2–layered $LiTMO_2$ structure. (b) Orthorhombic 80 atom conventional crystallographic unit cell of ribbon-superstructure O2–$Li_{1.2}Mn_{0.8}O_2$.

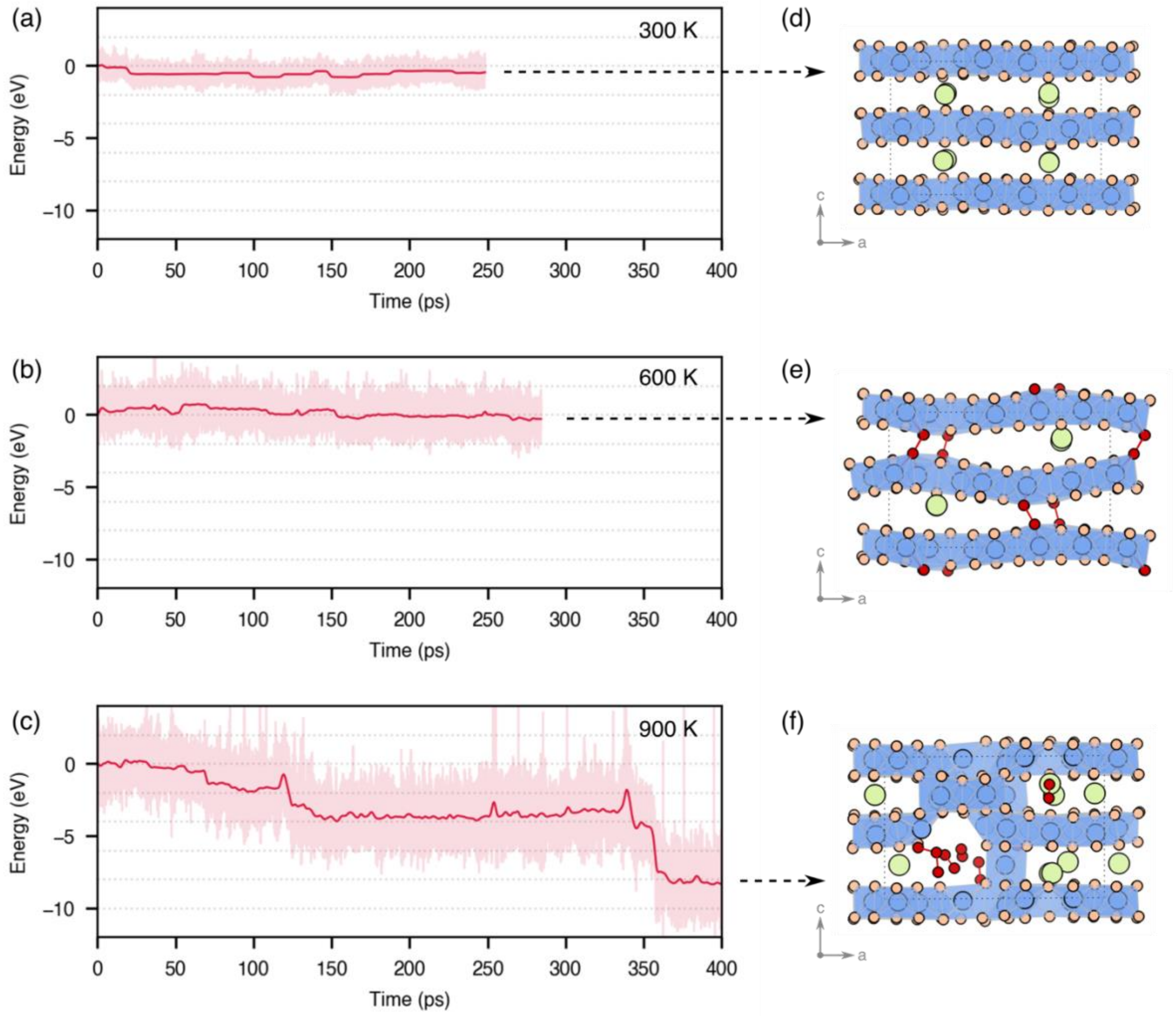

Supplementary Figure 2. (a–c) DFT+U total energy from AIMD trajectories of ribbon-superstructure $Li_{0.2}Mn_{0.8}O_2$, run at 300K, 600K and 900K. The shaded region shows the range of the fluctuations of the total energy. (d–f) The final structures from the trajectories, fully-relaxed with HSE06+D3. In the 300K simulations (a), no O–O dimerisation occurred (d) after ~250 ps. In the 600K simulations (b), interlayer O–O dimerisation occurred, but there was no Mn migration (e) after ~280 ps. In the 900K simulations, O–O dimerisation and Mn migration occurred in the first 100 ps. Some Li^+ ions have been removed for clarity.

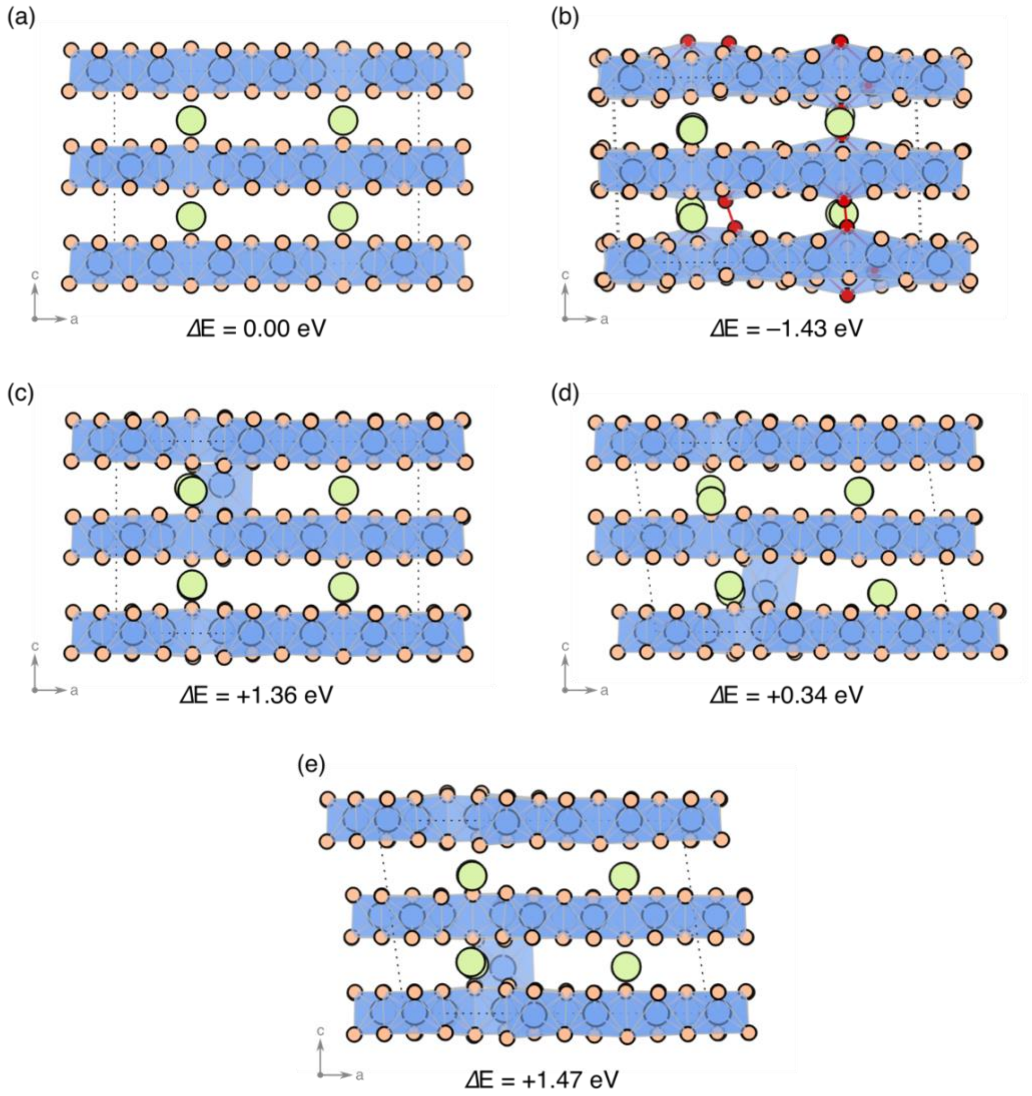

Supplementary Figure 3. Relaxed geometries and relative energies $Li_{0.2}Mn_{0.8}O_2$ structures with Mn in interlayer sites, compared to the pristine ribbon structure, and the interlayer dimer structure relaxed from the AIMD simulations. (a) Pristine ribbon superstructure (structure I in Figure 2, main text). (b) Interlayer dimer structure from the AIMD trajectory (structure II in Figure 2, main text). (c–d) Structures with Mn in interlayer sites.

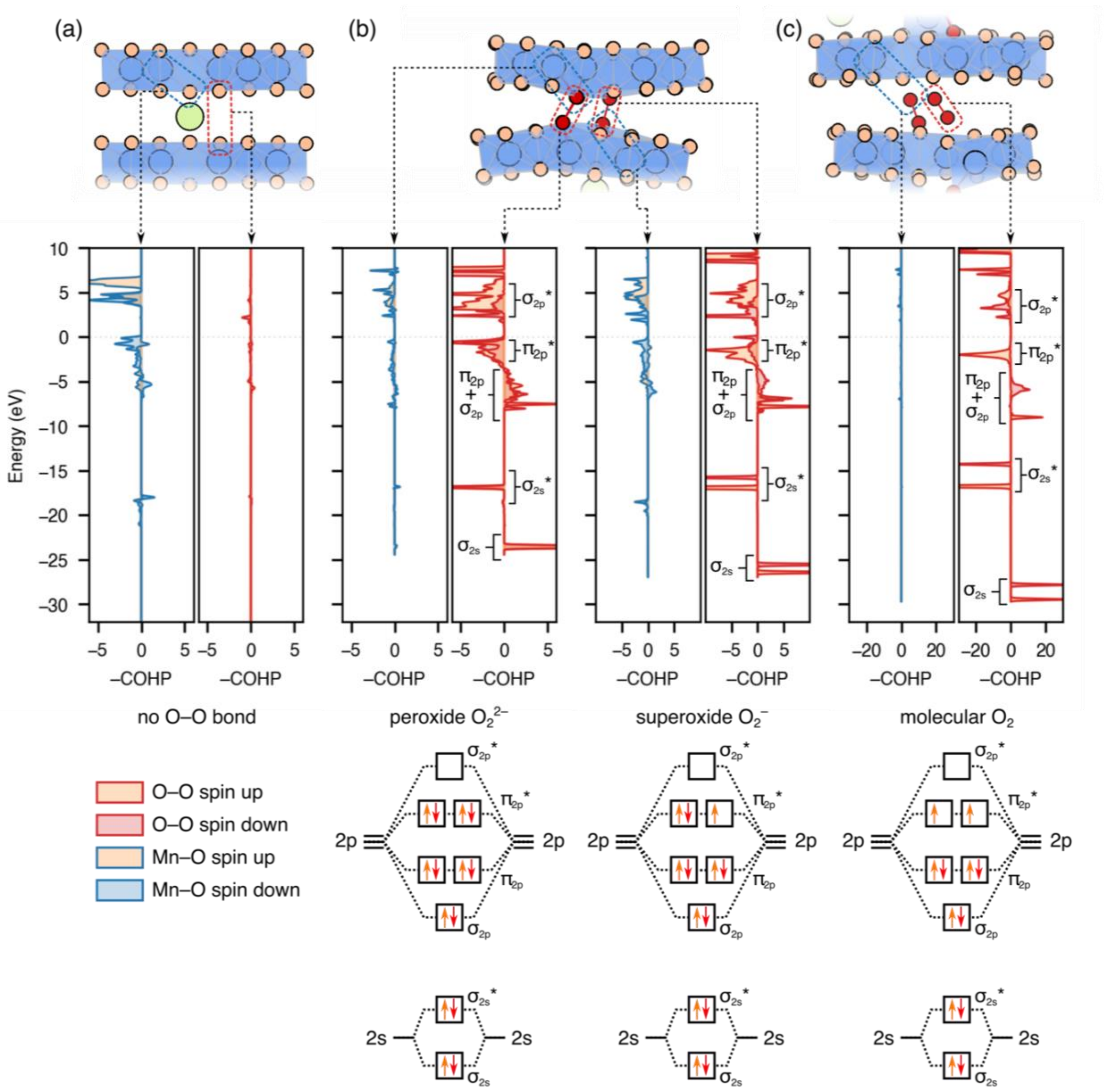

Supplementary Figure 4. Crystal orbital Hamiltonian population (COHP) analysis of Mn–O and O–O bonding changes during the structural rearrangement mechanism identified by 900K ab initio molecular dynamics simulations. Structures being analysed were extracted from the AIMD trajectory and fully relaxed with HSE06-D3, before being analysed. Structures (a–c) are structures I, II and III from Figure 2, Main Text. (a) In the pristine-delithiated structure, the Mn–O atoms have a bonding overlap, whilst there is almost no overlap between the two O atoms. (b) When the peroxide and superoxide species form between the layers, the Mn–O bonding is reduced, whilst there is now some bonding between O atoms. (c) When O_2 molecules form, there is almost no overlap between Mn and O, indicating no bonding. The O atoms display characteristic bonding overlap for a ground-state triplet O_2 molecule.

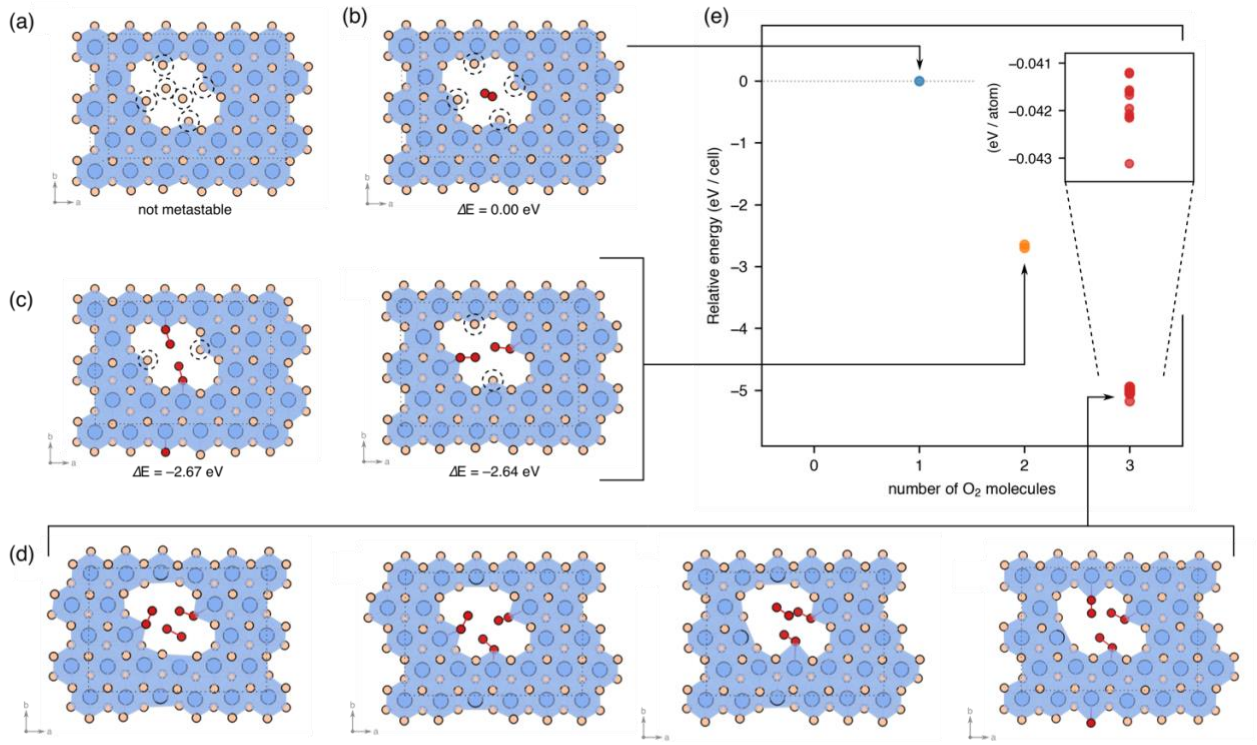

Supplementary Figure 5. Structures that map to the same lattice configuration (i.e., have the same cation configuration) that contain a different number of O–O bonds, or a different arrangement of the O_2 molecules. (a) A mapped lattice structure with all atoms on their lattice sites. All structures in (b) to (d) map to this lattice. Dotted circles identify O–X_6 sites and O–MnX_5 sites (where X sites are cation vacancies). This lattice structure is not a local minimum on the potential energy surface: the O–X_6 sites in the centre of the void always spontaneously dimerise to form an O_2 molecule. (b) Relaxed structure obtained from the dimerisation of the two O–X_6 sites in (a). This structure (b) contains four O–MnX_5 sites (circled in dotted lines), which favour dimerisation. The structure is metastable; there is an activation barrier for the O–MnX_5 sites to dimerise. (c) and (d) Structures in which the O–MnX_5 sites have dimerised to form one and two more O_2 molecules respectively. Each time two O–MnX_5 sites dimerise to form an O_2 molecule, the structure lowers its energy by ~2.5 eV, highlighting that the energy of the lattice structure (a) is minimised when the number of O–X_6 and O–MnX_5 sites is maximised. (e) Energies of structures in panels (b) – (d). All structures in (d) maximise the number of O–O bonds in the structure, map to the same lattice and fall within a 3 meV atom^{-1} energy range, meaning that any structure can be used to train the cluster expansion.

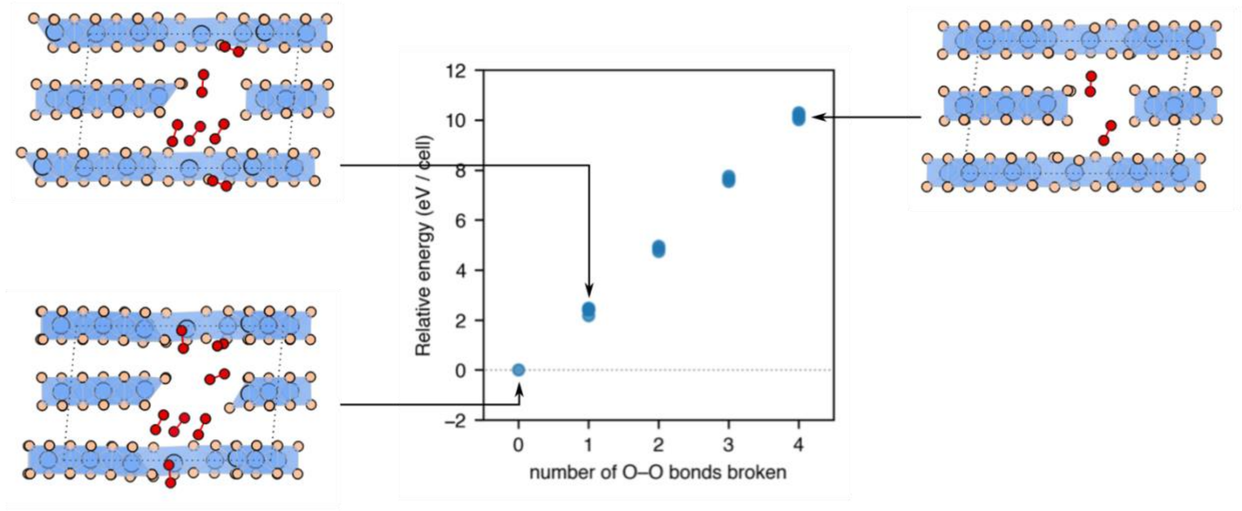

Supplementary Figure 6. Energies of a structure which have the same cation configuration but different numbers of O–O bonds. The structures were obtained by consecutively breaking the O–O bonds and manually-recombining the O atoms with undercoordinated Mn ions to form O–MnX_5 sites (where X denotes the number of cation vacancies around the O site). The energy goes up each time an O–O bond is broken, by approximately 2.5 eV, making it thermodynamically unfavourable for O_2 species to dissociate and re-combine with Mn.

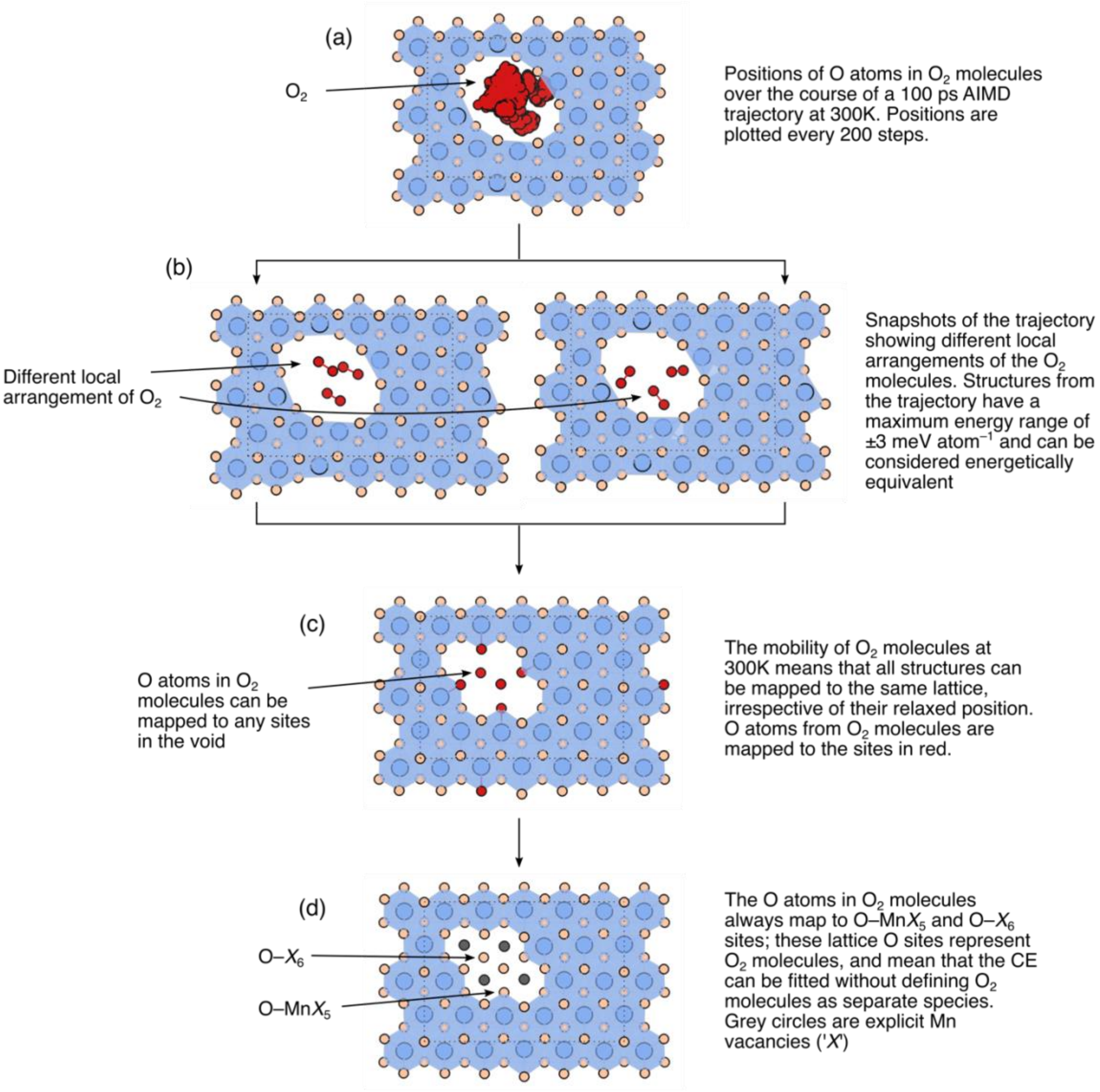

Supplementary Figure 7. Illustration of how the room-temperature mobility of O_2 molecules means that all possible rotational and translational configurations of O_2 molecules can be represented in a lattice model by the same configuration. (a) Position of O_2 molecules over the course of a 100 ps AIMD trajectory at 300K. O_2 molecules can freely rotate and translate to access all positions in the void. (b) Snapshots of the trajectory showing the positions of the O_2 molecules. All snapshots, when fully-relaxed with HSE06, have a maximum energy range of ±3 meV atom^{-1} and thus can be considered energetically equivalent for the purpose of training the cluster expansion. (c) All possible O_2 configurations can be mapped to the same lattice configuration. O atoms from O_2 molecules are mapped to specific sites (in red). (d) The O atoms in O_2 molecules are always O–MnX_5 and O–X_6 sites.

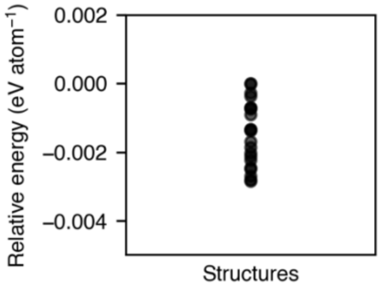

Supplementary Figure 8. Energies of 20 Structures with the same cation configuration and number of O–O bonds, with different local arrangements of those O–O bonds, fully relaxed with HSE06. The maximum range of energies is 0.36 eV/cell, or ~3 meV/atom in the 120 atom cell.

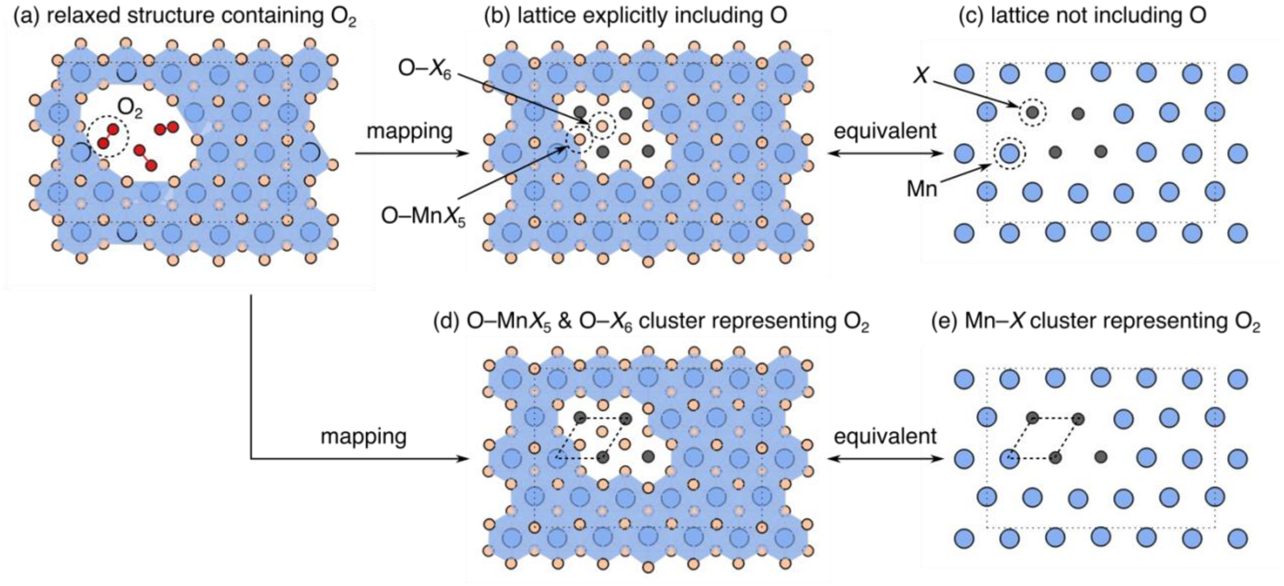

Supplementary Figure 9. Lattice structure both including the O sublattice (a), or omitting it (b). Both lattice configurations are equivalent, because there is no disorder on the O sublattice in (a), meaning that it is redundant. In (a), O_2 molecules are defined by O–MnX_5 and O–MnX_6 sites. In (b), the same information is contained in the clusters of Mn (blue circles) and cation vacancies (*X*, black circles). Thus, the description of O_2 molecules arises implicitly from the cation clusters.

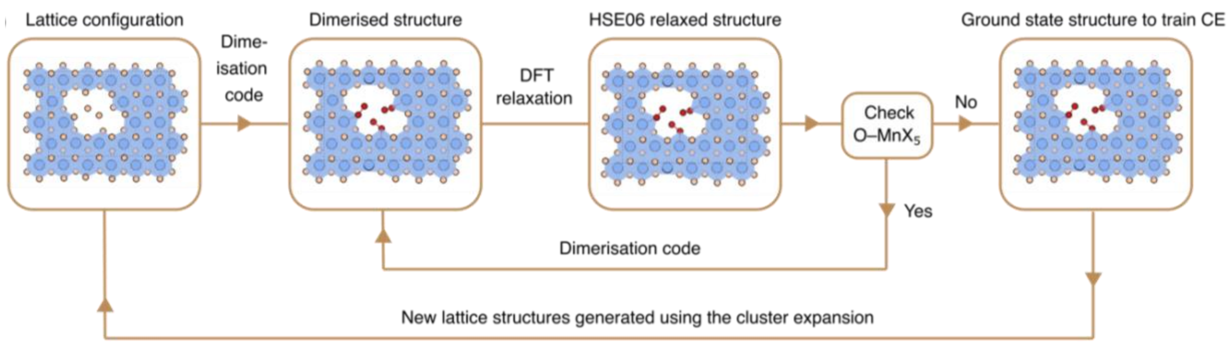

Supplementary Figure 10. Schematic showing the strategy for determining the lowest-energy relaxed configuration for a given lattice configuration. We constructed a piece of structure-manipulation code using *Pymatgen* that automatically dimerises all O–MnX_5 and O–X_6 sites, to give, at no computational cost, and good approximation for the geometry of a low-energy structure. The structure was then fully relaxed. This code was applied to lattice configurations generated iteratively using the cluster expansion and Monte Carlo simulations to efficiently establish ~630 low-energy structures with which to train the final cluster expansion Hamiltonian.

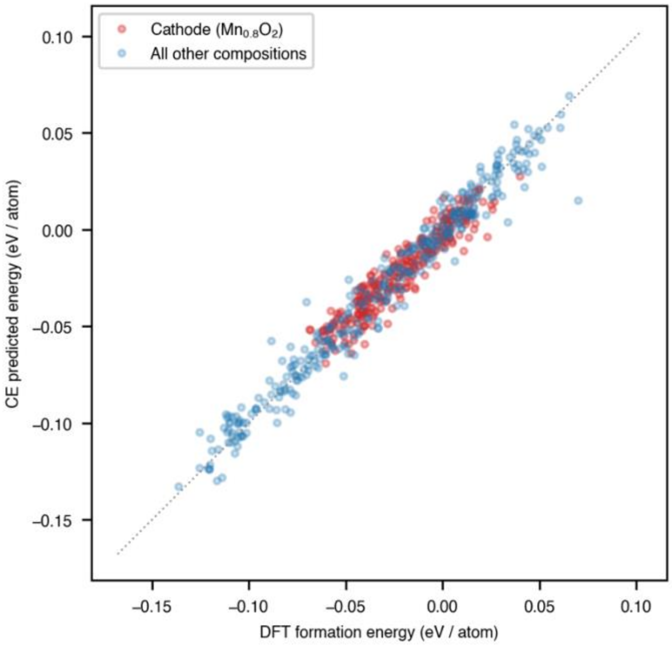

Supplementary Figure 11. Comparison of DFT calculated energies of mapped structures used to fit the cluster expansion vs. predicted energies from the cluster expansion for the same structures.

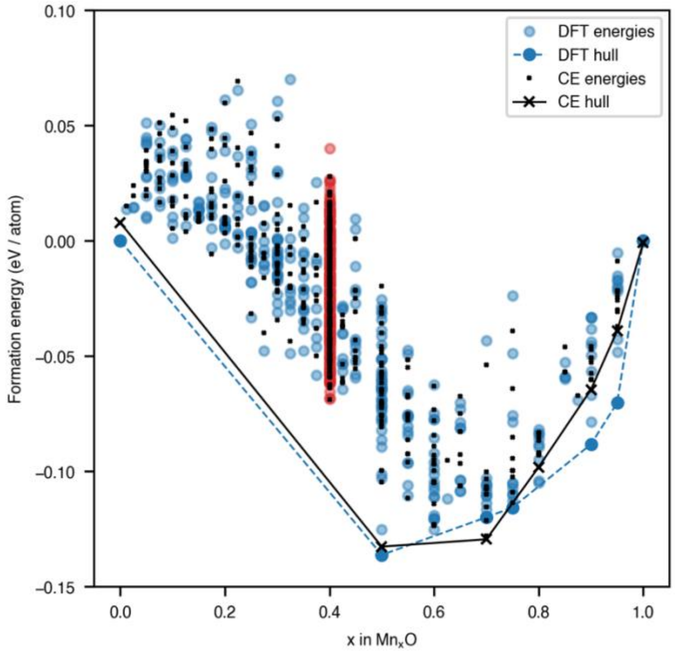

Supplementary Figure 12. DFT-calculated ground-state convex hull, compared against the convex hull predicted from the cluster expansion. Red data points are the cathode composition ($Mn_{0.8}O_2$), and blue points are all other compositions.

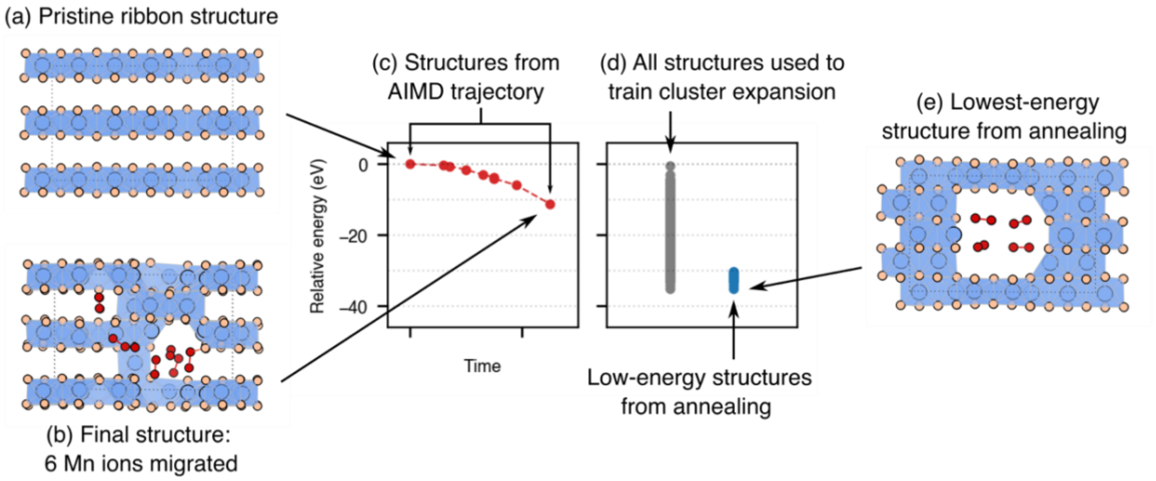

Supplementary Figure 13. (a) Fully relaxed pristine ribbon structure before AIMD simulations. (b) Final structure from the AIMD simulations, showing 6 Mn ions that have migrated to new sites. (c) Energies of structures from the AIMD simulation. (d) Energies of al the structures used to train the cluster expansion. (e) Lowest-energy structure from the CE-driven MC annealing simulations. All structures were fully relaxed with HSE06.

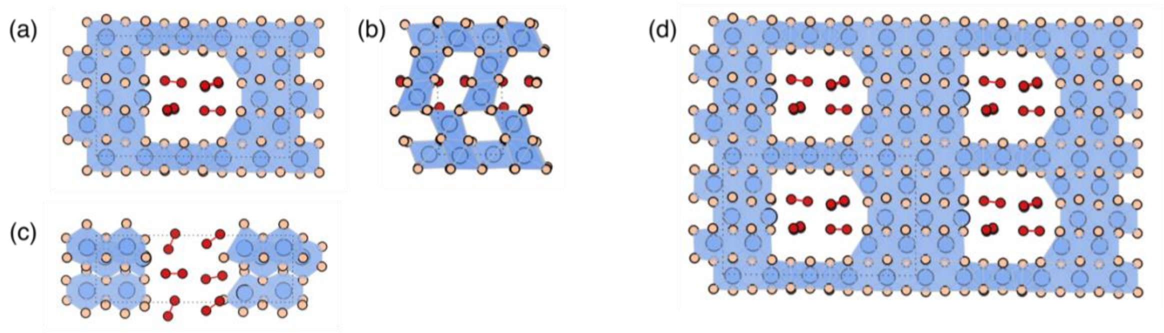

Supplementary Figure 14. Relaxed of the lowest-energy configuration generated by the cluster expansion. Projections along the *a* direction (a), *b* direction (b) and *c*-direction (c). (d) Shows the structure in a (2×2×2) expansion of the unit cell. The structure features MnO_2 like regions, with no face-sharing of Mn polyhedral, and small voids containing O_2. Although the cell is too small to truly phase-segregate, the formation of a small void mirrors the phase segregation observed in larger cells.

References

1. Sanchez, J. M., Ducastelle, F. & Gratias, D. Generalized cluster description of multicomponent systems. *Phys. Stat. Mech. Its Appl.* **128**, 334–350 (1984).

2. Van der Ven, A., Thomas, J. C., Puchala, B. & Natarajan, A. R. First-Principles Statistical Mechanics of Multicomponent Crystals. *Annu. Rev. Mater. Res.* **48**, 27–55 (2018).

3. Barroso-Luque, L. *et al.* Cluster expansions of multicomponent ionic materials: Formalism and methodology. *Phys. Rev. B* **106**, 144202 (2022).

4. Vinckeviciute, J., Kitchaev, D. A. & Van der Ven, A. A Two-Step Oxidation Mechanism Controlled by Mn Migration Explains the First-Cycle Activation Behavior of Li_2MnO_3-Based Li-Excess Materials. *Chem. Mater.* **33**, 1625–1636 (2021).

5. Chase, M. W., Jr. NIST-JANAF Thermochemical Tables. *J. Phys. Chem. Ref. Data Monogr.* **9**, 1019−1951 (1998).
